# Tracing Prescribed Knowledge Flows in Wastewater Management Policies: An AI-Assisted, Governmentality-Informed Framework with Insights from Indonesia

**DOI:** 10.1007/s00267-025-02277-0

**Published:** 2025-09-22

**Authors:** Roald Niels Christiaan Leeuwerik

**Affiliations:** 1 Leibniz Centre for Tropical Marine Research (ZMT), Bremen, Germany; 2 Institute for Geography, University of Bremen, Bremen, Germany

**Keywords:** Policy analysis, Wastewater management, Knowledge flows, AI, Governmentality

## Abstract

Policy documents allow for the study of prescribed knowledge flows in decision-making processes. Although policy documents have been analyzed previously in wastewater studies, a more systematic approach to analyze prescribed knowledge flows remains to be developed. Guided by governmentality, this article proposes a framework to investigate prescribed knowledge flows and gain insights into intended stakeholder roles, techniques and technologies used to govern, as well as the nature of knowledge that should be exchanged. The framework is built upon the new possibilities by Artificial Intelligence (AI) by developing a prompt to identify prescribed knowledge flows. Building on an analysis of Indonesian policies, the study presented in this paper illustrates how a decentralized and community-led approach for wastewater management is planned. The approach intends to drive behavioral change and community-led management initiatives, thereby protecting public health and environmental quality. However, challenges include scarce details on prescribed stakeholder roles and an incomplete operationalization of national and/or regional provisions. While verification of AI output remains necessary, AI support saves time by reducing the need for full-text reading and summarization of identified prescribed knowledge flows. The method described in this paper can be used by decision-makers to facilitate critical inquiry of policies, or by non-governmental stakeholders to better understand complex legal texts and opportunities for involvement in decision-making.

## Introduction

Many environmental issues, such as wastewater pollution, occur in contexts where stakeholders have different knowledge about severity and appropriate solutions. However, different kinds of knowledge are often not equally used for decision-making. Differences reflect power asymmetries, with decisions often shaped by the knowledge of more powerful groups. Power asymmetries can lead to the reinforcement of existing inequalities, with marginalized groups particularly carrying the burden of environmental pollution. Moreover, they can hinder the implementation of effective management strategies when these are poorly adapted to local needs, or lack acceptance by end users (Deelder 2013; Karpouzoglou 2012; Karpouzoglou and Zimmer 2016; Zimmer 2012).

The study of transfers of knowledge between stakeholders—*knowledge flows*—can offer a perspective to understand power relations (e.g., Buechler and Mekala 2005; Muñoz-Erickson and Cutts 2016; Stott and Huq 2014). In the context of wastewater, studies have compared expert and non-expert knowledge types. Different methodologies have been employed, including interviews, focus group discussions, workshops, and participant observation. Notably, non-expert knowledge tends to be excluded, in part due to the relatively closed nature of decision-making processes as well as the reliance on specific engineering, scientific, or legal perspectives (Leeuwerik et al. 2025).

Another way through which knowledge flows have been investigated is document analysis. For example, policy documents can be analyzed to determine how knowledge is supposed to flow. In wastewater studies, policy documents have been examined to elucidate historical and/or contemporary provisions pertaining to community participation (Getachew 2013; Hahn et al. 2023; Mahoney 2011; Nare and Odiyo 2013) and the ways through which wastewater issues are framed (Kedzior 2011; Sutherland et al. 2015; Zimmer 2012). Moreover, some studies have concentrated on the roles assigned to government actors or Community-Based Organizations with regard to management implementation (Al’Afghani et al. 2019; Sutherland et al. 2015; Zimmer 2012). While these studies offer valuable insights, such as the importance of providing complete descriptions of knowledge flows where senders and receivers are clearly established (e.g., Abeysuriya et al. 2019; Juric 2018; Putri and Moulaert 2017), a more systematic way to analyze prescribed knowledge flows is still lacking.

Nevertheless, regulatory frameworks are highly relevant by mediating the kinds of knowledge that are considered for decision-making. Therefore, a critical inquiry in prescribed knowledge flows may not only serve to improve transparency of decision-making processes but can also enable stakeholders to better understand their roles or to advocate for a stronger involvement (Leeuwerik et al. 2025). In this article, an AI-assisted method to identify prescribed knowledge flows in policy documents is presented. To demonstrate application of the method, results obtained from a study of key Indonesian wastewater policies will be shared.

## Methodology

This study is informed by the concept of *governmentality*. Foucault introduced governmentality after his historical analysis of the shift from sovereign power, focused on territorial control and domination over individuals, to the emergence of bio-power which manages populations’ well-being through regulation and support rather than direct coercion. Foucault refers to this process as the governmentalization of the state, wherein the conduct of populations is shaped through various forms of knowledge, scientific disciplines, norms, and institutions (Davidson et al. 2008, p. 317; Dean 2010, pp. 58–59; 118–120; Foucault 2007, pp. 104; 108–110; 367). It is through these configurations that governmentalities, referring to rationalities on *how to govern*, are formed. Governmentalities are often taken for granted, with their foundation resting upon a body of knowledge, theories and philosophies that have been developed over time, giving shape to *regimes of practices* (Dean 2010, pp. 18–19; 24; 27) These regimes of practices are structured ways of acting and reflecting, encompassing the techniques and mentalities that inform how conduct is shaped (Dean 2010, pp. 38; 49–50). Dean (2010) distinguishes between *four dimensions* that together constitute a regime of practice. It is important to consider these dimensions together, as changes in one affect the other.

One dimension concerns the *forms of identity* of those governing and governed, including expected types of conduct and the kind of transformations aimed for (Biza et al. 2022; Dean 2010, pp. 43–44; 49–50; 217; Zimmer 2012). In practice, such distinctions exert a significant influence on how individuals perceive themselves and others, while also demonstrating how governments attempt to control people by having them identify with specific descriptions (Dean 2010, pp. 43–44). For wastewater management, stakeholders are often divided into sub-groups in need of different kinds of interventions based on their existing practices. For example, scholars such as Biza et al. (2022), McFarlane (2008) and Putri (2019) show how divisions between the (colonial) elite and poorer (native) communities have long-lasting consequences. Poorer communities were seen as undisciplined and undeserving leading to an uneven coverage of sanitation services. This continues to reinforce structural inequalities and leads to measures such as forced displacement and tightened supervision to force behavioral change.

Another dimension is the *techne of government*, which considers the techniques and technologies to accomplish rule (Dean 1996, 2010, p. 42; 44; 217; Flannery and McAteer 2020; Rose and Miller 2010). This dimension has shown a tendency to assume different meanings with fluid distinctions between techniques and technologies of government (e.g. Dean 1996; Rogers et al. 2016; Sheng et al. 2022; Zimmer 2012). To speak of technologies of government, Dean (1996) proposes different thresholds. Firstly, technologies can be made up of greater assemblages of techniques of government to guide conduct. An example of this would be the implementation of horizontal eco-compensation alongside environmental tax incentives and public awareness campaigns to encourage sustainable river basin management (Sheng et al. 2022). Secondly, techniques can integrate in larger socio-technical systems such as those for production, consumption and communication where different technical objects, monetary flows, communication networks, human actors etc. are involved. Rather than in isolation, techniques are then part of and interact in a larger system. An example would be a system of income support for the unemployed, where someone receives unemployment allowances (based on eligibility checks and verification procedures), as well as training and other necessary services to find new employment (Dean 2010, p. 39). Another way through which technologies can manifest is when forces are generated that are qualitatively different from the augmentation or synthesis of existing forces, resulting in new forms of power. For example, public health measures taken during the Covid-19 pandemic, such as quarantine zones, face mask obligations and travel restrictions, demanded an infrastructure of enforcement, coordination, communication and logistics for implementation (Van Dijk et al. 2022; Zweig et al. 2021). Lastly, techniques of government must be connected to requirements of performance to make them calculable, measurable and comparable and to ensure conduct is effectively guided to achieve specific objectives (Dean 1996, 2010, p. 196; 223; 225; Zimmer 2012).

Dean (2010) distinguishes four categories of technologies of government, namely technologies of agency, citizenship, discipline and performance. These technologies are summarized in Table 1.

**Table 1:** The four categories of technologies of government

Technology of government	Purpose	Examples
Technology of agency	Includes ways through which individuals and authorities are guided to take responsibility (Dean 2010, p. 223; 225; Swyngedouw 2005; Zimmer 2012).	Wastewater issues are often attributed to a lack of active engagement or inability of stakeholders to contribute to management. Different strategies can be implemented, such as decentralization, privatization of wastewater services and awareness-raising campaigns (Dean 2010, p. 196; Kyessi 2005; Lippi et al. 2008; Zimmer 2012).
Technology of citizenship	Aims to foster capacity of citizens to contribute to societal development through techniques of self-esteem, empowerment and consultation in activities like community development, environmental impact assessments and health promotion campaigns (Cruikshank 2019; Dean 2010, p. 83; 196; 225; Swyngedouw 2005). Intersects with agency: while citizens should respect state authority, this technology promotes the image of a “good” citizen who exercises agency (Cruikshank 2019).	By creating an image of a “good citizen” through norms of citizenship, people form an understanding of their rights and duties in society. Examples include promoting the use and maintenance of installed latrines (O’Reilly and Budds 2023; Zimmer 2012, pp. 47–48), and Community-Led Total Sanitation (CLTS) which emphasizes behavioral change and taking own responsibility without relying on state intervention (Galvin 2015).
Technology of discipline	Disciplinary measures aim to directly guide conduct. Is connected to technologies of agency and citizenship as it can help to ensure their proper implementation (Dean 2010, pp. 130; 144; 182–183; 202).	Could for example involve AI-supported tracing of industrial water pollutants to their source (Wang et al. 2019), surveillance by sanitary inspectors (Zimmer 2012, p. 184), or imposing fines on polluters (Raff and Earnhart 2018).
Technology of performance	Involves the use of parameters against which assessment can take place. Aims to ensure efficiency and foster accountability. Connected to other technologies by helping to ensure people are empowered or exercise their agency in the correct manner (Dean 2010, p. 223; 225; Swyngedouw 2005).	May for example involve the definition of reductions in wastewater discharges to be achieved (Zimmer 2012, p. 46), audits to monitor the operations of (privatized) service providers (Dean 2010, p. 196), or the application of AI models to predict the performance of wastewater treatment plants (Nourani et al. 2018).

Where the previous two dimensions have focused on identities as well as the means to accomplish rule, two other dimensions of governmentality are more specifically concerned with the knowledge that shapes a regime of practice.

The dimension of *ways of seeing* looks at how a governance issue is defined in terms of the problems that must be solved. This can be expressed in representations like maps, media articles and scientific reports (Creech 2020; Dean 2010, p. 41; 206; 217; Lemke 2009; Rogers et al. 2016; Rose and Miller 2010; Tazzioli and Walters 2016). Policy documents, owing to their prescriptive nature, provide a traditional source of data for governmentality studies (McKee 2009). In the context of wastewater management, the fact that pollution is not necessarily experienced similarly or considered equally problematic is important. Due to different experiences and perceptions, certain wastewater-related practices or forms of pollution may be problematized more than others. These differences, in a power-laden context, ultimately affect the knowledge that enters the decision-making process and influences how the issue is defined (Zimmer 2012).

Once an issue is defined, different kinds of knowledge can be used to establish truth. This refers to the dimension *ways of knowing* (Dean 2010, p. 42; 206; 217; Rogers et al. 2016; Sheng et al. 2022). With its focus on establishing specific forms of truth, this dimension is closely related to Foucault’s power-knowledge nexus (Foucault 1980, pp. 51–52; 131; Mehta et al. 2007) where regimes of truth establish what information is right or erroneous. In practice, this means that the production of knowledge has to adhere to certain rules or fixed procedures to be accepted (Karpouzoglou and Zimmer 2016; Rogers et al. 2016; Sheng et al. 2022). In wastewater management, a clear manifestation is the assessment of water quality, where choices made about indicators to be used influence the understanding of decision-makers and the actions they take (Karpouzoglou and Zimmer 2016; Zimmer 2012).

## Methods

This section is intended to facilitate comprehension and reproduction of the presented method. First, the context of the study, materials used and operationalization of the four dimensions for regimes of practice will be presented. Following this, the section will outline the strategy for policy analysis and data recording.

### Study Area

**Fig. 1:**
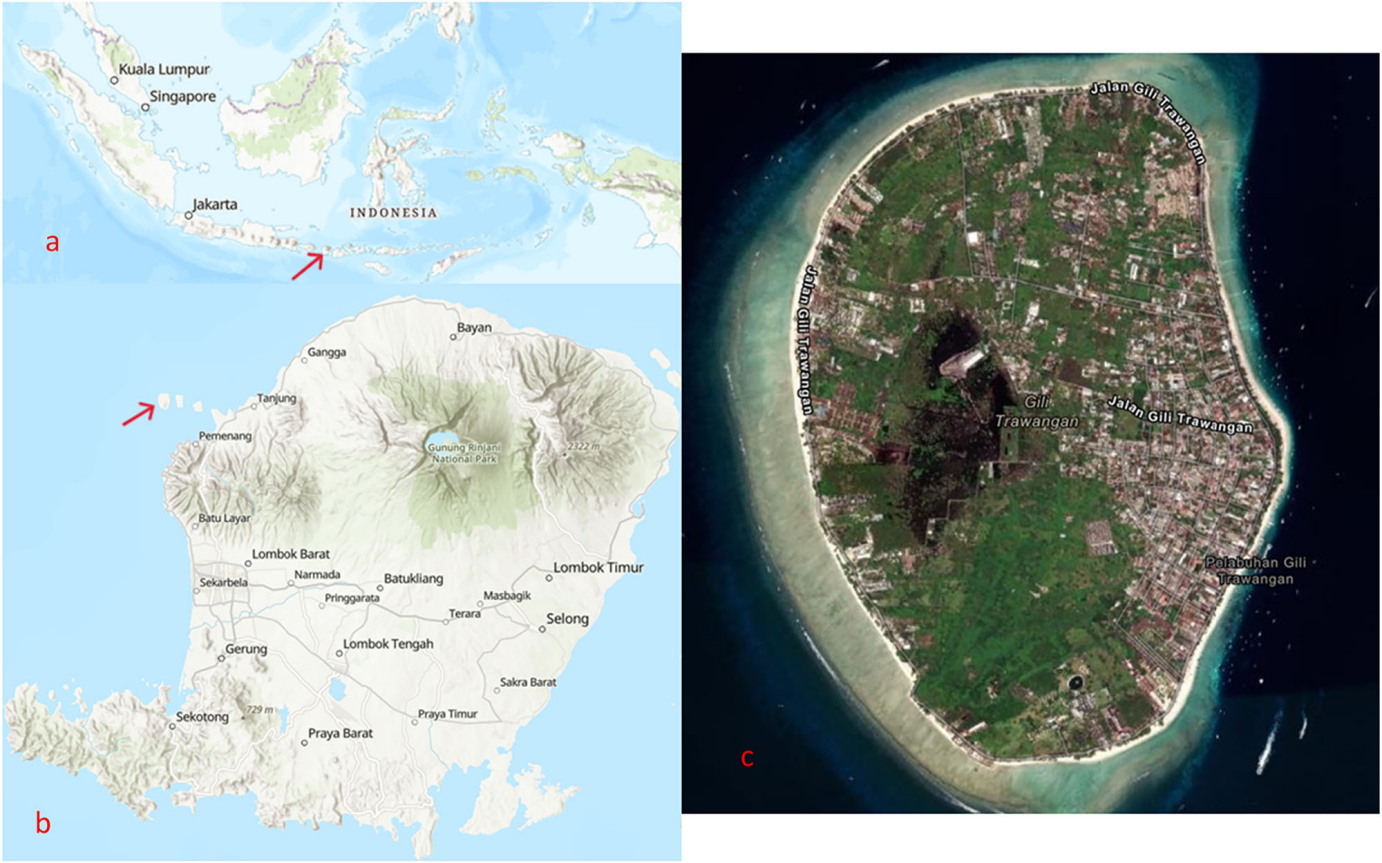
Location of Lombok in the Indonesian archipelago (**a**); the island of Lombok with the location of Gili Trawangan (**b**); the island of Gili Trawangan (**c**). Maps were obtained from ArcGIS: https://www.arcgis.com/apps/mapviewer/index.html

This article is part of the TransTourism project, which looks at wastewater issues on tropical islands. Coastal regions around the world are densely populated and vulnerable to wastewater pollution. Particular challenges exist for islands due to their remote location complicating infrastructure development. In the tropics, these challenges are often exacerbated through rapid growth of the tourism sector (Crisman and Winters 2023; Reopanichkul et al. 2010; Wells et al. 2016). An example of an island that has encountered such challenges is Gili Trawangan, situated off the coast of Northwest Lombok (Indonesia) (Fig. 1).

Gili Trawangan measures 6 square kilometers and is situated in a marine park with neighboring islands Gili Meno and Gili Air. The island has witnessed a rapid development of the tourism sector from the early 1990’s. Just prior to the Covid-19 pandemic, the island welcomed around a million tourists per year. Marine tourism provides an essential source of livelihood for the around 2000 permanent residents of the island (Partelow et al. 2023; Partelow and Nelson 2020). Out of a shared interest for the establishment of a top-class diving destination, the first dive shops formed informal institutions with traditional local leaders. This has led, among others, to the development of public infrastructure and services like roads, waste collection, schools and medical services. However, the influx of domestic and foreign investors has reduced the influence of informal institutions and limited their ability to respond to sustainability challenges (Partelow 2021; Partelow and Nelson 2020). The current situation has led among others to wastewater pollution. An important reason is that infrastructure development has stayed behind the increasing number of inhabitants, tourism businesses and visitors. Although the national government has funded the construction of a decentralized sewage system for part of the island, this system is not yet operational and effectively managed. Therefore, most of the wastewater is disposed of through open-bottom septic pits from which the effluent seeps into groundwater and the sea (Dodds et al. 2010; Hampton and Jeyacheya 2015; Partelow et al. 2023; Partelow and Nelson 2020). Pollution from wastewater has led to poor water quality, with scores well below standards for marine life and marine tourism, posing risks such as higher coral mortality, algal overgrowth and the incidence of waterborne diseases (Kurniawan et al. 2023).

**Fig. 2:**
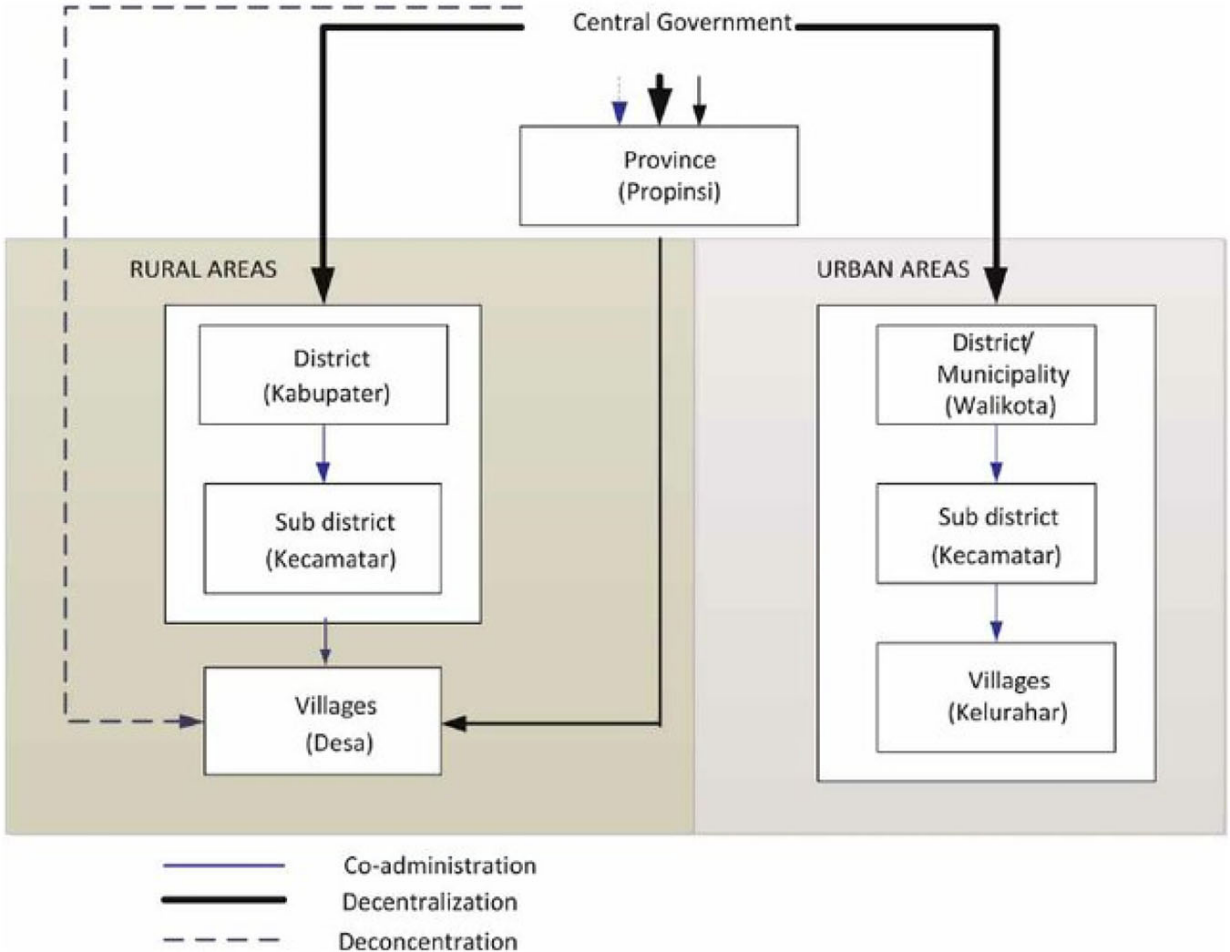
Decentralized government structure of Indonesia (image obtained from: Simanjuntak et al. 2012)

The limitations of informal institutions led to demands for more formal arrangements with the North Lombok regency and West Nusa Tenggara province (Partelow and Nelson 2020). In Indonesia, a process of decentralization has been initiated by the year 1999 (Holtzappel and Ramstedt 2009). In this process, authority has gradually been transferred from the central government to regional governments (Fig. 2). Authority is concentrated at the level of districts, which are regencies in rural areas (kabupaten) and municipalities in urban areas (kota). Provinces, above these levels, have a coordinating function to manage issues across regencies and municipalities. Within regencies and municipalities there are sub-districts (kecamatan), which are further divided into village governments in rural areas (pemerintah desa) and wards in urban areas (kelurahan). Regencies and municipalities are now responsible for, among others, public works, health, education and environmental affairs (Efriandi 2021; Mamma 2016; Shoesmith et al. 2020; Simanjuntak et al. 2012). As a further testimony to decentralization stands the level of the village government, which is presided by a village head and supported by a village council. This level has gained even more authority by law in 2014 which gives it a degree of autonomy over higher government levels. This is expected to empower local communities and to improve financial management by direct funding from the national government (Shoesmith et al. 2020).

### Study Materials and Operationalization of Four Dimensions

The material for the policy analysis encompasses a range of national and regional policy documents, varying from wastewater-specific policies to broader environmental policies. Policy documents were identified through internet searches, the study by Waluyo (2021), and through contact with local government officials. All policy documents have been translated into English using Google Translate.

To guide the analysis of prescribed knowledge flows in policy documents, it was decided to focus on the policy formulation and revision stages of the policy-making process. Another focus for analysis included the preparatory processes required for the implementation of specific projects such as infrastructure development (e.g., environmental impact assessments). This choice was made due to the fact that these stages typically involve intense stakeholder interactions (e.g., Dupont et al. 2024; Makwambeni et al. 2023; Singh et al. 2020; Theokritoff and Lise D’haen 2022).

For the four dimensions for regimes of practice, further decisions for operationalization were made (Table 2).

**Table 2:** Operationalization of the four dimensions for regimes of practices

Dimension	Operationalization
Forms of identity	Included under this dimension are knowledge flows prescribing rule-making authority, and knowledge flows prescribing broader responsibilities for governmental and non-governmental stakeholders in the formulation and implementation of wastewater management.
Techne of government	In essence, technologies encompass different techniques (Dean 1996). Based on identified knowledge flows as part of forms of identity, technologies can be discerned. Technologies of citizenship focus on community engagement and empowerment, technologies of agency look at the level of authority granted to various actors, technologies of discipline emphasize structured systems of control, and technologies of performance focus on accountability and evaluation.
Ways of seeing	With its focus on the representation of an issue, of specific interest were prescribed knowledge flows to manage wastewater impacts. The following impacts were identified in scientific literature: • Environmental—impacts from wastewater on biodiversity and health of terrestrial, coastal and marine environments, affecting not only natural habitats but also human livelihoods (e.g. Kocasoy et al. 2008; Reopanichkul et al. 2010). • Health-related—direct and indirect ways through which wastewater affects human health; including water-borne disease transmission and groundwater contamination (e.g. Getachew 2013; Rusca et al. 2022). • Socio-cultural—wastewater management and community dynamics; including space usage as well as the degree of alignment with local cultural values and needs (e.g. Cairns 2014; Mueller et al. 2020).
Ways of knowing	For this analysis the focus has been on prescribed knowledge flows describing the development of water quality standards and indicators. Such standards and indicators play a key role in assessing wastewater pollution and guiding actions that are taken.

### Identifying and Recording Prescribed Knowledge Flows

**Fig. 3:**
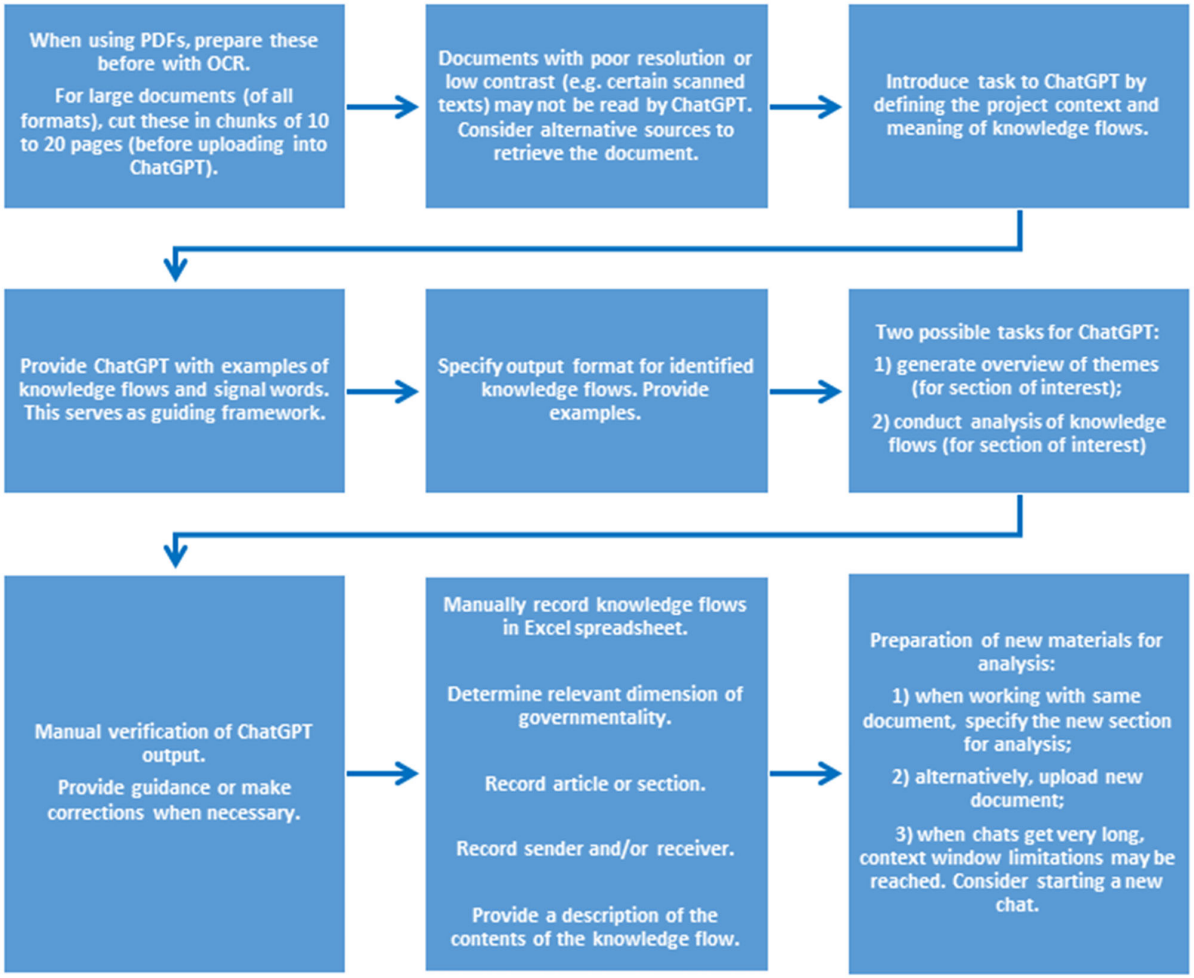
Flow diagram of the policy analysis supported by ChatGPT

For analyzing prescribed knowledge flows, the new opportunities provided by AI, especially *ChatGPT*, have been leveraged. For this research, the models GPT-4 and 4o were used. To structure the analysis, a prompt was developed based on a first round of manual reading of policy documents. This included law 32 (2009) and law 23 (2014). While the structure of the prompt will be described, references will be made to the full prompt in the supplementary materials. A general overview of the process is visualized in the flow diagram below (Fig. 3).

The prompt starts with a brief introduction (Part 1—step 1 and 2), clarifying the focus of the analysis and providing a definition for knowledge flows. Following that (Part 1—step 3), a document with examples of knowledge flows is shared (Supporting document: examples of knowledge flows). This step helps ChatGPT to understand the focus of the analysis and to provide more tailored output. Importantly, examples of *complete flows* (sender-receiver relationship), as well as *incomplete flows* (only a sender or receiver is specified) are provided. Here, the initial full manual reading of policy documents proved valuable by providing material for the document with examples of flows. Additional flows were added as the policy analysis progressed to provide for a variety in examples.

In the next step of the prompt (Part 1—step 4), a document with *signal words* commonly associated with knowledge flows is shared (Supporting document: signal words for knowledge flows). The first version contained words identified during manual reading of policy documents. Further signal words were added during the policy analysis as they were identified by ChatGPT or during manual verification of output. Importantly, the prompt clarifies that the list is not meant to be exhaustive. This allows ChatGPT to identify alternative signal words, yet it provides guidance (Giray 2023).

Coming at this point, the prompt outlines the preferred format to describe knowledge flows and gives examples to ensure ChatGPT provides consistent output (Part 1—step 5) (Giray 2023). The examples were based on initial output from ChatGPT which allowed to compare format styles. Importantly, it clarifies the need to identify individual knowledge flows, applicable for cases where more knowledge flows can be encountered in a single piece of text (e.g. a legal article). It also instructs to indicate the specific location (e.g. article) where the knowledge flows are discussed as well as to avoid inferring or deducing stakeholders when these are not directly cited. Providing such specifications and limitations helps to obtain output that aligns with the particular needs for analysis (Grabb 2023; Lee and Shin 2024).

After these introductory steps have been completed, the policy document that should be analyzed can be uploaded to ChatGPT. The prompt includes two main tasks for ChatGPT based on particular needs. The first task is to generate an overview of themes that are covered in particular sections of interest (e.g. in a sub-chapter or a range of articles) (Part 2). Examples of theme descriptions are provided to instruct on preferred format. The second task is the analysis and description of prescribed knowledge flows (Part 3). The specific part of the document to be included for analysis is specified, and the instructions emphasize that output should be similar to the format style as previously outlined. Importantly, it also reminds ChatGPT to avoid inferring or deducing the roles of stakeholders that are not directly cited.

To develop the prompt and improve output, it was iteratively tested and refined by comparing ChatGPT output with results of initial manual analysis and by manually scanning analyzed texts during the rest of the analysis (Knoth et al. 2024; Lee and Shin 2024). Scanning involved looking for keywords and pieces of information in the analyzed policy document based on ChatGPT output to verify alignment. In case of differences, it involved a closer reading to determine the nature of the differences and trying to correct ChatGPT (Part 4). The prompt was also tested for different document types; for example, not all policy documents would use the traditional structure of individual articles. Some policy documents may, for example, use sections and sub-sections with text paragraphs. The prompt takes this into account by including instructions for these different documents.

During analysis, identified prescribed knowledge flows were manually recorded in an Excel file. Firstly, the position within the policy document was indicated and the sender(s) and/or receiver(s) were noted. After recording this information, the nature of the knowledge had to be considered. If this was related to any of the impacts considered under ways of seeing, a short description of the flow was included under the respective category. If it related to the formulation of water quality standards or the selection of indicators, the description was included under ways of knowing. In case policies were found to include knowledge flows that describe stakeholder identities (i.e., prescribed roles or responsibilities) in wastewater management, these were included in a separate spreadsheet. In all cases, an additional column was provided for including any additional remarks (e.g. personal observations, possible relations to previous articles or policy documents).

## Results

This section will start with a reflection of using ChatGPT for policy analysis. Following this, the results from the analysis of prescribed knowledge flows in Indonesian policy documents are described through the four dimensions for regimes of practices. It should be noted that this section does not attempt to be exhaustive but aims to illustrate the kinds of insights that can be obtained. It does so by specifically considering the *key policies* on wastewater management; that is those policies that form the basis for wastewater management in Indonesia.

### Experiences with ChatGPT to Support Policy Analysis

The most significant benefit of using ChatGPT for this policy analysis was that it helped to save time. The amount of time saved depends on the size of the policy document and sections of interest therein, and the experience of the analyst with the method. In this study, some smaller policy documents of 20 to 40 pages (e.g., no. 3 of 2014; no. 16 of 2008) have been fully analyzed within 3 hours, while larger environmental laws of over 100 pages (e.g., no. 32 of 2009; no. 22 of 2021) with 150 to 200 pages of potential relevance have been analyzed within 3 working days. This then included having ChatGPT generate an overview of themes for sections of potential interest. Most significantly, the time saved was achieved by reducing the need for manual full-text analysis and identification and summarization of prescribed knowledge flows.

Nevertheless, manual verification of output proved necessary to iteratively refine the prompt and to provide ChatGPT guidance. As the analysis progressed and the prompt was refined, in many cases output by ChatGPT could be directly used to record prescribed knowledge flows after verification. In other cases, more detail might be preferred or ChatGPT needs to be corrected to obtain adequate output (see part 4 of Supplementary Materials for instructions). For example, an issue that was encountered on a few occasions was that ChatGPT misidentified a range of legal articles or a section of text, and provided incorrect output. Such instances are illustrative of a challenge with AI called *hallucination*. This means the AI presents erroneous, fabricated or incomplete results confidently as if they are correct. A common cause for this is that the training data of the AI model does not match with the task at hand (Janéafik and Dusek 2024; Kamel 2024). However, another cause could be poor quality of uploaded files for analysis such as PDFs (e.g., bad scans, formatting issues, OCR errors) (Liu et al. 2024; Piryani et al. 2025). Therefore, PDFs of good quality are preferably used to reduce risks for hallucination (Adhikari and Agarwal 2024). During the analysis, converting PDF files to Word was found to be effective in case of persistent incorrect output when using PDFs. The risk for hallucination may also increase when uploading and analyzing larger documents at once. Although ChatGPT can sometimes refer back to earlier sections to identify prescribed knowledge flows when a full document is uploaded, dividing large policy documents into smaller parts (up to 20 pages) helps reduce the risk of hallucination (e.g., Ibrahim et al. 2025).

Another challenge in the earlier stages of analysis was to have ChatGPT identify more than one prescribed knowledge flow in a single article or section of text. This was addressed by providing ChatGPT with more complex examples, both in the supporting document with examples of knowledge flows as well as in the examples for preferred output (part 1—step 5 of the prompt). However, the possibility that senders or receivers of knowledge are overlooked remains in more complex texts. When this is observed, ChatGPT can be instructed to analyze in more detail specific articles or sections where senders or receivers were missed (see part 4 of Supplementary Materials).

### Forms of Identity and Techne of Government

The analysis of prescribed knowledge flows has provided insights in stakeholder roles (forms of identity) and techniques and technologies of government (techne) in wastewater management. An overview of results is provided in the table below (Table 3).

**Table 3:** Overview of stakeholder roles and techniques and technologies of government in key wastewater policies

Law	Stakeholder roles (forms of identity)	Techniques and technologies (techne)
Government regulation 38 (2007) *^1^—distribution of government affairs between the government, provincial regional government, and regency/city regional government Law 23 (2014) *^1^—on regional government administration *^1^: while these policies are not specifically focused on wastewater management, they are foundational for the determination of responsibilities of local governments in Indonesia. Both include articles on wastewater management.	These policies establish wastewater management as a “mandatory affair”, which has to be implemented at central, provincial and regency/city levels (art. 4 & 7 of 38 2007, p. 6–7; 8–10) (art. 11 & 12 of 23 2014, p. 11–12).	*Technologies (of agency and citizenship)* A nested governance structure exists where regencies/cities are responsible for the implementation of local regulations in line with provincial and national standards. Provinces are involved as regional coordinator, evaluator of performance and supervisor of implementation (appendix of 38 2007) (art. 344, 349, p. 182, 184 & appendix—table C—of 23 2014). The national government establishes minimum service standards and overarching strategic frameworks (art. 18 of 23 2014, p. 16). Chapter 14 on society participation (art. 347, 354 of 23 of 2014, p. 183, 186-187) encourages regional governments to inform community groups and organizations about policy implementation and to promote their involvement through capacity development. A structured approach for community involvement in planning, budgeting, implementation, monitoring and evaluation is recommended. *Techniques* To enhance public service management, regional governments (provinces, regencies/cities) may establish communication fora involving the community and related stakeholders. Public services management includes (1) implementation of services; (2) management of public complaints; (3) information management; (4) Internal monitoring; (5) outreach to the community; (6) consulting services; and (7) other public services in accordance with statutory provisions (art. 345 of 23 2014, p. 182-183).
Ministry of public works regulation 16 (2008)—development of residential wastewater management systems	This law describes Community-Based Total Sanitation (STBM), which requires the involvement of the national and regional governments, as well as the community. Strategies will target five areas: (1) increasing access to infrastructure; (2) increasing the role of community and businesses; (3) development of legal regulations (national and regional); (4) strengthening institutions; (5) increased financing (chapter 2, chapter 4).	*Technologies (of citizenship)* STBM (Community-Based Total Sanitation) aims to increase the role of community and business stakeholders. The main objective is to improve coverage of central treatment or on-site systems. Important ways to achieve this are to increase public understanding, encourage participation, increase business investment potential, and support formation of community self-help groups through training and assistance (chapter 4).
Ministry of health regulation 3 (2014)—Community-Based Total Sanitation	Main national STBM regulation that establishes the five pillars to be used by central and regional governments as well as by communities in the implementation of STBM. These pillars are intended to break disease transmission and include: (1) stop open defecation; (2) wash hands with soap; (3) management of drinking water and household food; (4) domestic solid waste management; (5) domestic wastewater management (art. 3, p. 4).	*Technologies (of agency)* Central government coordinates STBM implementation and provides technical training to lower government levels. Provinces ensure coordination across sectors and regencies, determine priorities and evaluate implementation. They also prepare training materials to be used by regencies/cities in training and coordination with officers and the community in their jurisdiction (art. 9, 10, 11, 12, p. 7–8). Universities, research institutes, businesses as well as government-contracted consultants can be involved in the implementation of STBM (at all governmental levels), which involves the preparation of training material, monitoring and evaluation of management initiatives, research and development and the provision of other materials necessary for implementation (art. 14, p. 9). *Techniques* A triggering workshop, described in the attachment, aims to educate and motivate communities to adopt better sanitation practices. During workshops, knowledge on wastewater risks and sanitary practices is shared by the STBM Facilitator Team and dialogue encouraged through questions on local sanitation issues. Community members are encouraged to observe and discuss sanitation practices and challenges and reach mutual understanding. Motivated members are encouraged to organize further planning sessions to improve sanitation (attachment). Sub-district/village/ward STBM Facilitator Teams, consisting of volunteers, community leaders, religious leaders, and supported by local village heads, should promote local STBM implementation (attachment).
Presidential regulation 185 (2014)—Accelerating the provision of drinking water and sanitation	A regulation that calls on national and regional governments to develop strategies for the development of the drinking water and basic sanitation sector (art. 7, 9, p. 4–5).	*Technologies (of agency and citizenship)* Requires the formulation of a National Drinking Water Roadmap and National Sanitation Roadmap. These national roadmaps drive the development of provincial roadmaps and regency/city sanitation strategies (SSKs in Indonesian). The development of regional roadmaps will be facilitated through the establishment of guidelines by the minister who administers government affairs in the field of sanitation. Local authorities will be assisted in preparation, implementation and monitoring and receive education and training to increase their capacity (art. 7, 9, 27–30, p. 4–5; 10–11). Community participation is described in article 37 (p. 12), stipulating the responsibility of regional governments to educate, advocate, socialize, promote and campaign to enhance community participation in drinking water and sanitation efforts. *Techniques* Article 15 (p. 8) stipulates the creation of *POKJAs*, defined as drinking water and sanitation working groups at provincial and regency level. These groups may consist of government officials, universities, professional organizations, community organizations, religious organizations, NGOs, media, businesses and community members.
West Nusa Tenggara governor regulation 78 (2023)—Five pillars of Community-Based Total Sanitation—Roadmap towards safe and sustainable sanitation years 2023–2026 North Lombok regency sanitation strategy 2022 - 2026 (SSK KLU—Strategi Sanitasi Kabupaten Lombok Utara 2022–2026)	These regional policies are implemented to fulfill the objectives stipulated in ministerial regulation 3 (2014) and Presidential Regulation 185 (2014). The sanitation strategy describes tasks of different regency offices and their relation with the community. BAPPEDA (planning agency) is responsible for preparing technical policies, coordinating regional development planning, and monitoring and evaluating policy implementation. The health office handles operational planning, supervision, and evaluation of public health services, including issuing hygiene sanitation certificates, promoting community health through advocacy and community empowerment, and overseeing environmental health policies. The department of public works, spatial planning and housing manages planning, implementation, and supervision of infrastructure, sanitation, and wastewater management while engaging communities through socialization and empowerment initiatives. The department of community and village empowerment prepares plans, supervises, and evaluates activities related to village administration, traditional institutions, and village-owned enterprises (BUMDes) while enhancing institutional capacity and promoting community welfare (chapter 2—table 2.10; appendix).	*Techniques* POKJA PPAS*^2^ working groups coordinate housing, settlements, drinking water, and sanitation implementation at provincial and regency levels. Regencies report on STBM implementation to the province, who reports to the ministry of health, ministry of public works and housing and BAPPENAS (planning agency). In the case of North Lombok regency, POKJA AMPL was involved in drafting the SSK. These working groups aim to unite government offices, community partners, academics, businesses, professional organizations, media, and interested community members to facilitate the objective of article 4(2) (p. 5), which is to align community-based total sanitation with local needs and wisdom (art. 5, 11 of 78 of 2023, p. 5-6; 8). *^2^: POKJA PPAS also includes housing and settlement concerns, in addition to the focus on water and sanitation by POKJA AMPL. Sometimes also referred to as POKJA AMPL/PPAS, or still as POKJA AMPL. However, their role is similar, serving as coordination mechanisms for water, sanitation and hygiene improvements.

For *^1^: while these policies are not specifically focused on wastewater management, they are foundational for the determination of responsibilities of local governments in Indonesia. Both include articles on wastewater management.

For *^2^: POKJA PPAS also includes housing and settlement concerns, in addition to the focus on water and sanitation by POKJA AMPL. Sometimes also referred to as POKJA AMPL/PPAS, or still as POKJA AMPL. However, their role is similar, serving as coordination mechanisms for water, sanitation and hygiene improvements.

**Fig. 4:**
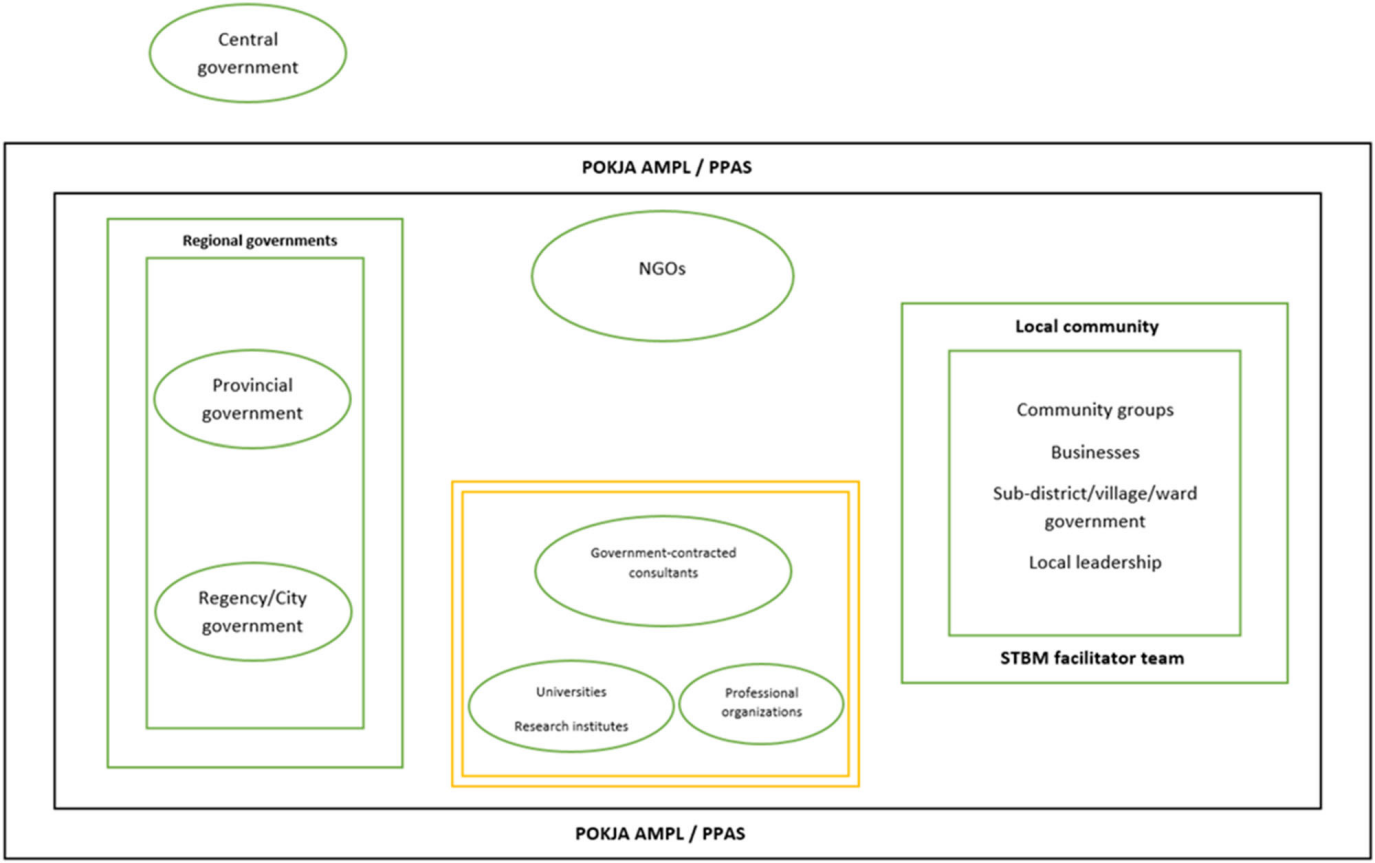
Stakeholders involved in Indonesian wastewater management

As Table 3 illustrates, the key policies emphasize technologies of agency and citizenship. A decentralized approach is pursued where regional governments are provided agency for wastewater management. Moreover, Indonesia wishes to improve wastewater management through Community-Based Total Sanitation (Indonesian abbr. STBM). It intends to increase the role of local communities by stimulating them to contribute to wastewater management through local facilitator teams (Al’Afghani et al. 2019; Galvin 2015). Furthermore, it is stipulated that working groups (POKJA) are to be established at provincial and regency level. Such working groups are an example of a technique of government meant to facilitate coordination between governmental and non-governmental stakeholders. The different categories of stakeholders and the composition of POKJA and STBM facilitator teams are visualized in the diagram below (Fig. 4).

To assess implementation progress, article 8 of ministerial regulation 3 (2014) (p. 7) determines that fully achieving the five pillars of STBM or one of the five individual pillars must be based on the assessment of a verification team (formed by the regency/city government). Only after official assessment can the sub-district, village or ward declare successful implementation and be provided an award by the governor (art. 7 of 78 Governor Regulation 78 (2023), p. 6-7). The appendices 1 and 4 of ministerial regulation 3 (2014) provide indicators to assess implementation. In general, these indicators could be described as:

- For STBM implementation—at least triggering has been done, individuals (natural leaders) or community groups take the responsibility to continue STBM interventions and prepare an action plan that is mutually agreed upon.
- Stop open defecation—only healthy latrines are used, no visible feces are found in the environment, sanctions or regulations or other efforts are put in place by the community for prevention, and a general monitoring mechanism is established by the community.
- Achievement of the 5 pillars of STBM—determined by verification team who monitors triggering and collects STBM data (e.g. community work plans, community activities).

### Ways of Seeing and Ways of Knowing

Concerning ways of seeing, key wastewater policies emphasize the management of health-related impacts. Ministerial regulation 3 of 2014 describes a triggering workshop (pp. 25–32) during which STBM facilitator teams are to motivate the community to reflect on local wastewater pollution, available sanitation facilities and sanitation practices. Motivated community members will be encouraged to develop local sanitation plans. To bring this forward, all government levels will raise awareness to increase community demand for improved sanitation. Regionally, governor regulation 78 of 2023, specifically article 6 (p. 6), and chapter 4.1 of the North Lombok regency sanitation strategy (pp. 149–152) emphasize the engagement of community and business stakeholders by the province, regency, sub-district, village or ward governments. Efforts aim at raising awareness on the importance of wastewater management for public health, increasing the role of *natural leaders* to encourage behavioral change, and to stimulate business investments in sanitation facilities.

Beyond public health, the approach further aims to address environmental and socio-economic impacts. For socio-economic impacts, the governor regulation 78 of 2023, specifically article 4(2) (p. 5), formulates the objective to implement management initiatives based on community knowledge and needs. Therefore, it is stipulated in chapter 4.1 of the North Lombok regency sanitation strategy (pp. 149–152) that the community actively contributes to the formulation of STBM policies at sub-district/village/ward level and provides suggestions to regional governments. Moreover, addressing open defecation and promoting the construction of sanitation facilities is considered an essential measure to improve environmental quality. This is among others mentioned in chapter 4.1 of the North Lombok regency sanitation strategy and in the triggering workshop description in ministerial regulation 3 (2014) (p. 25), where the facilitator should emphasize how improved wastewater management contributes to a clean environment and helps to avoid waterborne diseases.

Concerning ways of knowing, no prescribed knowledge flows were identified in the key wastewater policies but only in broader environmental policies. Although these policies are not the focus for this article, these flows will be briefly characterized due to the relevance of water quality standards and indicators to assess wastewater pollution. Indonesian legislation, specifically article 157 of government regulation 22 (2021) (pp. 72–73), establishes a public right to receive information about water quality protection and management plans that must be formulated by regional governments and the national government. Moreover, the public has a right to advocate for and to participate in water quality monitoring and improvement initiatives, and to complain about water pollution. Another form of community engagement is described in article 161 (p. 74), where regional governments and the national government are given the task to facilitate partnerships between the community and businesses to reduce water pollution.

Other prescribed knowledge flows detail interactions between business or project owners and regional governments, stipulated in government regulation 22 (2021) and ministerial regulation 68 (2016). Article 4 of ministerial regulation 68 (2016) (pp. 5–6) obliges any person involved in an activity that generates domestic wastewater to treat wastewater and report treatment results to the regency/city and province. Articles 135–137 (pp. 64–65) and 249–258 (pp. 104–109) of government regulation 22 (2021) detail obligations to request a permit for activities that result in wastewater discharges to fresh and marine water bodies. Further prescribed knowledge flows include horizontal and vertical intergovernmental flows. Article 10 of ministerial regulation 68 (2016) (p. 8) obliges regency/city governments to report to the province and national government on domestic wastewater management, including water quality inventories and installed treatment technologies. This information is used to calculate water pollution load capacities and trade allocations, facilitating the setting of regional standards. Further intergovernmental flows, established in government regulation 22 (2021) articles 118–120 (pp. 57–58), involve the formulation of regional and national water quality protection and management plans, which serve to ensure a coordinated approach to protect water resources from pollution.

### Potential Challenges for Implementation

**Fig. 5:**
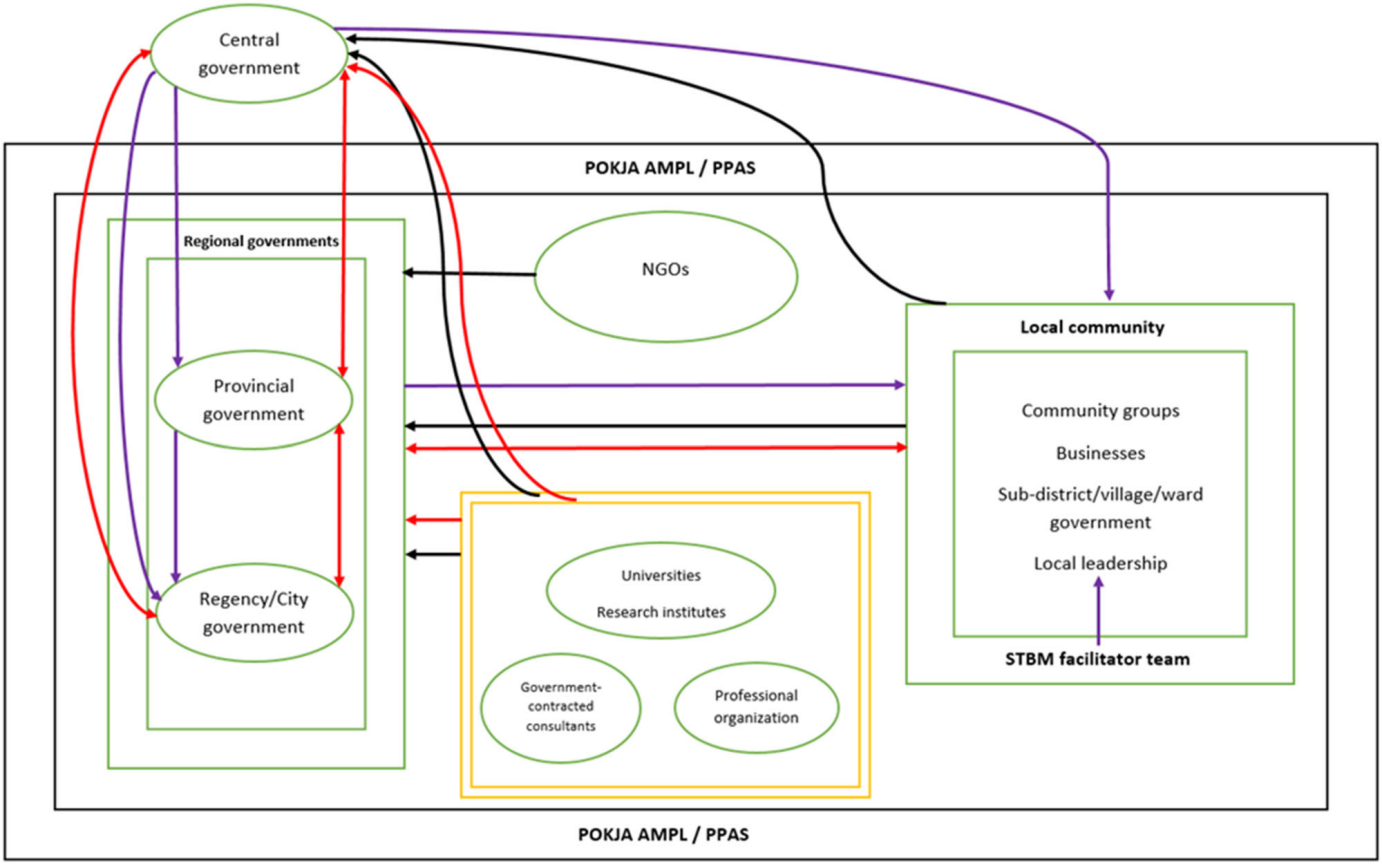
Diagram with stakeholders and prescribed flows between them. Black arrows refer to policy-making; red arrows represent policy coordination, permitting, monitoring and supervision; and purple arrows represent capacity-building and awareness-raising

The analysis of the four dimensions for regimes of practice revealed a decentralized and community-based approach for wastewater management. There are prescribed knowledge flows that emphasize awareness-raising and capacity building, while others focus on policy-making or look at permitting, intergovernmental coordination and evaluation of policy implementation. A general overview of prescribed knowledge flows between stakeholders is visualized in the diagram below (Fig. 5).

The prescribed management approach requires cooperation and coordination between community stakeholders and regional governments. In this context, a critical review of prescribed knowledge flows revealed some challenges. An overview of these challenges has been included in the table below (Table 4).

**Table 4:** Overview of challenges for the implementation of wastewater management in Indonesia

Prescribed knowledge flow	Potential obstacle(s)
A fundamental requirement for stakeholder participation by all government levels and across policy domains is established in law 23 (2014) (art. 354, pp. 186–187). Regionally, a key provision for stakeholder participation in wastewater management specifically is included in governor regulation 78 (2023) (art. 6, p. 6).	While these policies establish key provisions, a challenge is their operationalization. Participation mechanisms, stakeholder access to information, and support given by the government for capacity building are not further detailed. A certain fragmentation can also be observed, with procedures for community participation in STBM to be stipulated through various national guidelines rather than through regional regulations as envisaged by law 23 (2014). These circumstances can make it challenging for stakeholders to understand their roles, participate meaningfully and to know which level of government to engage with and when.
For the development of public services and wastewater management, different kinds of coordination mechanisms are foreseen. Ministerial regulation 16 (2008) promotes the formation of “community self-help groups” (chapter 4). Furthermore, law 23 (2014) provides the possibility for establishing “communication fora” (art. 345, pp. 182–183). Presidential regulation 185 (2014) (art. 15, p. 8) further prescribes the formation of POKJAs (working groups). Regionally, a key provision on POKJAs is included in governor regulation 78 (2023) (art. 5, pp. 5–6).	A challenge is that participatory mechanisms are often more generally described, making their implementation harder. Already in ministerial regulation 16 (2008), the potential members of the prescribed community self-help groups and the kinds of knowledge to be exchanged are not further detailed. Similarly, their degree of authority in the implementation of management is not further addressed. For POKJAs, a particular challenge is that their functioning is not specified in detail. In particular, duties and work procedures are not further clarified in national and regional legislation despite this being prescribed. This can complicate the implementation of these working groups, and the equal participation by different stakeholders. In a similar vein, existing legislation does not further specify what “communication fora” for public services development should entail in terms of their members, the kinds of knowledge to be exchanged or by whom this should be further regulated.
Wastewater management should be a concerted effort. Regional governments, comprising of provinces and regencies/cities, should contribute to wastewater management implementation. There is an emphasis on awareness-raising to enhance community participation and accelerate service provision, as laid down in Presidential regulation 185 (2014) (art. 37, p. 12). Furthermore, the community is not merely a participant, it should play an active role in management formulation. An objective that is also established in the North Lombok regency sanitation strategy (chapter 4.1).	While awareness-raising is deemed crucial, legislation does not further detail the kinds of knowledge to be transmitted to the community. Moreover, the respective roles for provinces and regencies/cities are not specifically described. While their roles and means of awareness-raising (e.g., triggering workshops) might be similar as under ministerial regulation 3 (2014), this is not directly specified as such. The sanitation strategy calls for the development of regulations and institutions at regency level, public-private partnerships for wastewater management, and an increased role of village/ward governments (including local regulations and village-owned enterprises—BUMDes). However, no further details on contents of regulations, stakeholders to be engaged, and the functioning and composition of management institutions are provided.

## Discussion

An analysis of prescribed knowledge flows for the four dimensions of regimes of practice in sections “Forms of Identity and Techne of Government” and “Ways of Seeing and Ways of Knowing” has shown that through technologies of agency and citizenship, regional governments and local communities in Indonesia are given key responsibilities for wastewater management. Wastewater policies focus on capacity-building and awareness-raising to drive community initiatives, while the assessment and management of water quality is regulated in broader environmental policies.

In section “Potential Challenges for Implementation”, challenges for the implementation of wastewater management have been presented. A complex policy framework is formed through references by national policies to regional regulations. Regional regulations are, however, not always sufficiently operationalized leading to a lack of specificity around aspects such as community participation and POKJAs. A complicating factor is that national policies themselves tend to offer limited guidance. While flexibility to adapt policies can be important for a vast country like Indonesia, regional governments face difficulties to implement policies due to limited resources (Abeysuriya et al. 2019; Al’Afghani et al. 2019). Moreover, existing studies note that legal ambiguities can pose a risk for shifting accountability. Government authorities may emphasize community responsibility while deflecting demands for enhanced public services (e.g., Deelder 2013; Kedzior 2011; Rusca et al. 2022; Singh 2017; Wessels 2023; Zimmer 2012). In order to determine where and how challenges arise, it is recommended to look further at actual implementation in different regions of Indonesia. Although a regime of practice can be identified from policies, it is not necessarily implemented in practice where stakeholders may interpret and implement legal provisions differently (Cruikshank 2019; Dean 2010, pp. 85–88; McAteer and Flannery 2025; McKee 2009; Zimmer 2012).

Concerning challenges for implementation, it is also important to distinguish cities, peri-urban and other densely populated areas (e.g., Gili Trawangan) from rural areas. While covered under the same legal framework (Abeysuriya et al. 2019; Putri and Moulaert 2017), the PPSP roadmap identifies solutions for underserved urban areas. These include individual improved septic tanks, or the implementation of neighborhood-scale treatment units referred to as “SANIMAS” (Community-Based Sanitation; e.g., communal septic tanks with simplified sewer lines) and “DEWATS” (decentralized wastewater treatment systems; e.g., by anaerobic reactors). For urban centers and commercial districts, the expansion or construction of sewer networks is planned. Meanwhile, affluent neighborhoods are often already covered by private septic tanks, sewer connections, or independent treatment systems (Colin 2011; CWIS TA-Hub 2021; Nisaa and Hajrah 2018; World Bank 2013). By contrast, in rural areas the focus is on the construction of basic facilities by the community itself to end open defecation and improve household toilet ownership (Cameron et al. 2019; Galvin 2015). Funding for rural and urban areas has been skewed, leading to improvised low-cost latrines and poorly constructed septic tanks or unsealed pits in rural areas (Cameron et al. 2019; CWIS TA-Hub 2021; Irianti and Prasetyoputra 2021; World Bank 2013). In contrast, significant resources have been allocated to urban areas by the central and regional governments as well as donor organizations. For example, 21.832 SANIMAS systems had been built by 2019, serving around 6 million people for an investment of about 1 billion dollars over two decades (CWIS TA-Hub 2021; Irianti and Prasetyoputra 2021; World Bank 2013). However, operating and maintaining these systems is an ongoing challenge as they rely on community management. Therefore, investments in technical support and facilitators are needed to ensure they are sustainably used (Partelow et al. 2023; World Bank 2013).

This research has illustrated a novel application of AI to assist in policy analysis. AI technology can facilitate analysis but it remains necessary to verify generated output (see section “Experiences with ChatGPT to Support Policy Analysis” for personal experiences). Apart from the issue of hallucination, there are further ethical reasons for careful verification. For example, with AI assisting in or taking over tasks from humans, it is important to clearly establish who is ultimately accountable for the results (Bouhouita-Guermech et al. 2023; Samuel et al. 2021). Another important consideration is that AI ethics are still in their infancy with standards not yet widely available and often lacking in clarity or practical applicability. This demands a careful approach by policy-makers, technology developers and researchers alike (Khan et al. 2022; Lee and Qiufan 2024, pp. 32, 209; Morley et al. 2023). These precautions notwithstanding, the use of ChatGPT in this research has saved time by reducing the need for full-text reading and manual summarization of prescribed knowledge flows. Since the main part of this analysis was conducted in the summer of 2024, new AI tools have emerged apart from ChatGPT, including Google’s NotebookLM and AI features integrated into qualitative analysis software such as NVivo and MAXQDA. A potential strength of these applications is their reduced risk of hallucination, as the AI remains grounded in the material uploaded by the user. By contrast, ChatGPT draws on a large general dataset which can lead to hallucinations when the model attempts to fill gaps or makes assumptions. On the other hand, these alternative tools may involve a steeper learning curve to navigate their features and workflows, while ChatGPT is highly accessible and low-key. The choice between them will therefore depend on individual preferences, as well as specific research needs. Given their respective strengths and limitations, and the continuing evolution of AI, further exploration of different tools for policy analysis is warranted.

## Conclusion

In this article, an AI supported policy analysis of prescribed knowledge flows has been described. Application has been demonstrated by focusing on wastewater management in Indonesia, illustrating how a decentralized and community-led approach is pursued while identifying certain challenges for implementation. The presented method can help decision-makers to identify potential legal weaknesses in descriptions of stakeholder engagement and knowledge exchange which could benefit policy revision. For non-governmental stakeholders, the method could facilitate a better understanding of complex legal texts and identify possibilities to become engaged in decision-making processes. The use of AI was found to save time by reducing the need for full manual reading and summarizing identified prescribed knowledge flows. However, critical verification of AI output remains necessary. Further application of ChatGPT and other AI tools in policy analysis is recommended to determine best practices and optimal use to meet different needs.

## Supplementary information

Supplementary information

## Data Availability

The prompt and supporting documents used in ChatGPT to identify prescribed knowledge flows in policy documents can be accessed through the Supplementary Materials.
