# Supplementary material for: Tracing Prescribed Knowledge Flows in Wastewater Management Policies: An AI-Assisted, Governmentality-Informed Framework with Insights from Indonesia
Source: Environ Manage. 2025 Sep 22;75(12):3461–78. doi: 10.1007/s00267-025-02277-0 (PMC12575479; doi:10.1007/s00267-025-02277-0)
Supplement: Supplementary file 1 — Supplementary information [file 267_2025_2277_MOESM1_ESM.pdf]

Tracing prescribed knowledge flows in wastewater management policies: An AI-assisted, governmentality-informed framework with insights from Indonesia

Supplementary file – prompt and supporting documents for knowledge flow identification using ChatGPT

Published in journal: Environmental Management

Roald Niels Christiaan Leeuwerik[1,2]

[1]Leibniz Centre for Tropical Marine Research (ZMT), Bremen, Germany
[2] Institute for Geography, University of Bremen, Bremen, Germany
E-mail: roald.leeuwerik@leibniz-zmt.de
ORCID: 0000-0002-8656-6253

Contents

As a note to users before 2
Part 1 (introducing the task) 3
Part 2 (generating an overview of themes) 7
Part 3 (analysis and description of knowledge flows) 9
Part 4 – asking further information, making corrections, providing guidance and particular tasks 10
Supporting document: signal words for knowledge flows 12
Supporting document: examples of knowledge flows 16

As a note to users before

ChatGPT can work with both PDF and Word files. With the GPT-4o model (launched May 2024) reading PDF files has improved but I found working with Word files may sometimes give better results. When encountering difficulties with PDFs (e.g. ChatGPT does not read correctly or hallucinates), a solution can be to convert a PDF file into a Word file and to try again.

In all cases, it is good practice to cut the analysis up in parts when working with ChatGPT. Also for smaller documents, it is recommended to go through it in parts (e.g. by individual chapters or ranges of articles). This is useful in any case as not everything will be of interest for analysis which can be ascertained from a first quick scan (e.g. table of contents or an inventory of main chapters). In case of doubt or when in need of a better understanding of contents, ChatGPT can be instructed to identify the themes that are discussed in a section of interest (see part 2).

When working with larger documents, I still found it hard to determine where to put the line in terms of maximum size to upload. Very large documents (e.g. 100 pages or more) are better uploaded in parts (e.g. by chapters or subchapters). But also smaller documents can give difficulties. I would estimate a good limit is around 20 to 25 pages. In case of large documents and extensive continued analysis of knowledge flows, the quality of responses may go down as the chat becomes longer (due to context window limitations). If this occurs, it is better to start a new chat and continue the analysis.

If possible (i.e. when documents are not too large) it can be beneficial to upload the document as complete. I found that ChatGPT is able to refer to previous or even later articles to identify senders or receivers.

Please always check the output generated by ChatGPT. ChatGPT may misidentify articles, identify senders or receivers that are not mentioned or overlook certain senders or receivers. Corrections can be made (detailed in part 4) and these are mostly effective, but AI tools are still in development. Make sure to at least scan the text ChatGPT has analyzed.

The majority of my work has taken place in the spring and summer of 2024. The development of AI and ChatGPT has continued since. Therefore, some of my notes have already been resolved by this time or new functionalities may change how we engage with these technologies. To illustrate, the addition of a "memory" which allows ChatGPT to retain specific instructions, as well as "projects" where supporting documents can be uploaded for ChatGPT to access for individual chats in the project, may make repetitive prompting redundant. Furthermore, context windows have expanded which allows for uploading and analyzing larger documents as a whole, and continuing chats for a longer time. Rather than a static document, the author therefore encourages those interested to experiment further and benefit of new functionalities that continue to be added.

A custom GPT based on the prompt is available under "GPTs" in the ChatGPT interface with the name "Knowledge flow identifier". Using this custom GPT allows for direct analysis of policy documents without first following all the steps under part 1. However, note that providing some background information on the focus and needs of analysis is still helpful for providing ChatGPT with context.

Part 1 (introducing the task)

Please note: the part below should not be skipped as it provides background for ChatGPT. It is told what is meant by "knowledge flows", gets examples to learn, is told how to recognize knowledge flows, and is instructed on preferred output.

Note that the contents of introductory parts 1 and 2 need to be edited according to specific research needs. The steps are numbered and should be uploaded one after another.

1 –

I need help analyzing policies for my research project. These policies encompass wastewater policies, as well as environmental standards and health standards with a broader legal coverage and validity (including wastewater).

The study sites for my research project are Indonesia, Zanzibar, and Colombia, where I am examining various forms of regulations, management plans, and strategies.

I am particularly interested in understanding the knowledge flows involved in the decision-making process. This includes interactions between diverse stakeholders within and outside of government in the process of preparing policies, plans or projects. Concerning knowledge, I am focusing on knowledge for the management of environmental, health-related and socio-cultural impacts used in decision-making.

Concerning knowledge flows, what is meant are exchanges of knowledge, either in a one-way or two-way exchange. Generally, knowledge flows are written in the form of sender – receiver relationships, where it is specified who gives knowledge to whom. However, in certain cases only the sender of knowledge or the receiver of knowledge will be specified. To aid in the identification of knowledge flows, a list of signal words will be provided to you later.

Please confirm once you have read this.

---

2 –

For the policy analysis of knowledge flows, I am focusing on the decision-making process. Typical activities during the decision-making process include:

- Identification of issues to be addressed by the policy: recognizing and understanding the environmental, socio-cultural, or health-related impacts that the policy, plan or project should aim to tackle.
- Determination of policy objectives: Clearly defining the goals that the policy, plan or project intends to achieve, which may include for instance environmental protection, prevention of disease transmission or prevention of adverse impacts from management on local communities.

- Assessment of potential impacts from the project or policy: Evaluating the potential environmental, health-related, and socio-cultural impacts that may result from the implementation of the proposed project or policy, such as pollution of local water bodies or community displacement.
- Identification of ways to mitigate potential impacts from the project or policy: Developing strategies and measures to minimize or offset adverse effects of the project on the environment, public health, and communities.
- Discussing alternative approaches to policy or projects: Exploring and considering different policy options and project designs to achieve the desired objectives.

Please confirm once you have read this.

Note: together with the instructions below, upload the document "Examples of knowledge flows"

3 –

First, I will share a document containing examples of knowledge flows identified in previously analyzed documents (Examples of knowledge flows). These examples are categorized into two types: complete knowledge flows and incomplete knowledge flows. Complete knowledge flows are characterized by both the sender and receiver of knowledge being explicitly defined. In contrast, incomplete knowledge flows may only specify either the sender or the receiver of knowledge.

The document begins with examples of complete knowledge flows, providing information on both the sender and receiver, followed by the example text. Similarly, examples of incomplete knowledge flows are presented, detailing information on either the sender or receiver, followed by the example text.

It is essential to understand that the examples provided are illustrative and not exhaustive.

Please ensure to identify only those knowledge flows where the senders or receivers are human entities such as people, organizations, or authorities. Do not consider non-human entities as senders or receivers in this analysis.

Confirm that you have read and understood these instructions and the document.

Note: together with the instructions below, upload the document "Signal words knowledge flows"

4 –

Before uploading the policy document, I now upload a PDF file with signal words that can help to identify knowledge flows in policies (Signal words knowledge flows). It contains signal words associated to senders and receivers of knowledge. Read these through.

Please note that this list is not meant to be exhaustive, you are allowed to identify other knowledge flows with different signal words. Please confirm once you have read this file.

---

5 –

Please conduct a meticulous examination of the document I will upload to identify and enumerate all instances of knowledge flow, focusing solely on explicitly mentioned senders and receivers. For cases where the knowledge flow is incomplete—meaning only the sender or receiver is directly specified—list that identified entity alone, explicitly noting the other as 'not specified.'

Within broader policy or strategy descriptions, pay close attention to individual statements or examples that demonstrate knowledge flows between entities. Each distinct instance, even if embedded within a larger narrative, should be identified and reported.

Avoid inferring or deducing the roles of any entities not directly cited in the text. If, after thorough analysis, no complete or incomplete knowledge flows are found in a specific section or article, clearly state that no knowledge flows were identified in that part. Enumerate each knowledge flow in the order they appear, adhering to the format provided below:

Format for Listing Knowledge Flows:

- **Article/Section Identified**: Indicate the specific article or section where the knowledge flow is observed.
- **Sender and Receiver**: State the sender and/or receiver as they are explicitly mentioned in the text. If one party is not mentioned, indicate this as 'not specified.'
- **Description(s) of Knowledge Exchange**: Provide a comprehensive description of the knowledge being exchanged, its purpose, and context, using complete sentences for clarity and thoroughness.

Example Entries:

- **Article 10**
  - Sender: Project proponent
  - Receiver: Local government
  - Description of knowledge exchange: The project proponent is mandated to submit an environmental impact study to the local environmental office, aiding the environmental permit application process.
- **Article 12**
  - Sender: Local community

- Receiver: Project proponent
    - Description of knowledge exchange: The local community is consulted by the project proponent to identify potential environmental and health-related impacts, which are then documented and processed in the permit application report.
- **Article 18**
    - Sender: Not specified
    - Receiver: Local communities
    - Description of knowledge exchange: Encouragement of local community involvement is emphasized to promote hygiene practices and enhance public health outcomes.
- **Article 43**
    - Sender 1: Person in charge of the Business and/or Activities
    - Receiver 1: Minister, Governor, or Regent/Mayor
    - Sender 2: Minister, Governor, or Regent/Mayor
    - Receiver 2: Public
    - Sender 3: Person in charge of the Business and/or Activity
    - Receiver 3: Public
    - Sender 4: Public
    - Receiver 4: not specified
    - Descriptions of knowledge exchange:
        - The Person in charge of Business and/or Activities submits an Environmental License Application (either DELH or DPLH), prepared using the Environmental Document Information System, to the Minister, Governor, or Regent/Mayor as per their authority.
        - The Minister, Governor, or Regent/Mayor makes the DELH or DPLH available to the Public via the Environmental Document Information System.
        - The Person in charge of the Business and/or Activity announces details about the business and its environmental considerations at the business location to the Public.
        - Following these announcements, the Public is invited to provide feedback within five working days. The receiver of this feedback (likely the Minister, Governor, or Regent/Mayor or Person in charge of the Business and/or Activity) is not specified.

If no knowledge flows are identified in a section or article, indicate the following:

- No knowledge flows were identified in [specify the section or article]. This indicates that the section does not explicitly describe the exchange of knowledge between defined entities or does not specify the entities involved in such exchanges.

Confirm when you have read this. Confirm that in case more knowledge flows can be identified, these can be listed as in the last example (article 43). I will then proceed to upload the document and provide further instructions.

This ends the introductory part. ChatGPT is now ready to analyze policy documents for knowledge flows. Different possibilities are provided in the following chapters.

Part 2 (generating an overview of themes)

Please note: For the descriptions of themes, ChatGPT is most useful to help understand larger sections. For smaller sections or sections with clear sub-titles, manual reading is generally sufficient. Knowledge flows can then be identified by skipping this part and using the instructions under part 3.

Please note: the prompt offers different possibilities, according to the structure of the document. Feel free to edit the instructions further according to specific needs or particular document structures. It is helpful to be as precise as possible when describing the document structure to ChatGPT.

The moment at which the instructions can be provided to ChatGPT is clearly indicated.

Please provide an analysis of the various themes presented in the document I have uploaded (WRITE DOCUMENT NAME). Themes within a legal context are often delineated by chapters, and may be further subdivided into different sub-chapters, parts or paragraphs. Please utilize these divisions, if provided, to identify and group sections that address similar topics.

Add the following phrase to the end in case of a traditional policy structure (edit when necessary)

Begin with the first article and continue until the final article as specified. List the themes in blocks of articles that pertain to the same subject matter. Please provide an overview of themes in chapter …
Focus on part / paragraph / section … (articles … to …)

Add the following phrase to the end in case of other document structures (edit when necessary)

Please provide an overview of themes in chapter … Focus on subchapter …

Continuation of the prompt (only required to add when asking for themes for first time in chat)

Subsequently, provide a concise description, ranging from 3 to 10 sentences, outlining the focus of the articles or sections encompassed within each theme. Keep the description of individual themes in one paragraph.

Examples to help ChatGPT structure (use according to document type)

In case of a traditional policy structure

Use the following structure:

- Articles 1 to 4. These articles cover general provisions, defining key terminology and explaining the rationale behind the policy. This policy focuses on environmental protection and provides provision on the process for environmental permits, control and prevention of environmental pollution and enforcement.
- Articles 13 to 19. These articles delve into the Environmental Impact Assessment process that needs to be conducted as part of the environmental permit. Procedural steps are outlined, together with roles and duties of different entities.

- Articles 13 to 14. These articles center on raising awareness among local communities regarding the potential risks of wastewater pollution. These initiatives aim at instilling a more critical attitude of community members towards environmental risks. The emphasis is placed on environmental protection and the prevention of health implications from pollution.

In case of other documents (adjust according to structure)

Use the following structure:

- Articles 1 to 4. These articles cover general provisions, defining key terminology and explaining the rationale behind the policy. This policy focuses on environmental protection and provides provision on the process for environmental permits, control and prevention of environmental pollution and enforcement.
- Chapter 3. This chapter delves into the Environmental Impact Assessment process that needs to be conducted as part of the environmental permit. Procedural steps are outlined, together with roles and duties of different entities.
- Sub-chapter 3.1. This sub-chapter centers on raising awareness among local communities regarding the potential risks of wastewater pollution. These initiatives aim at instilling a more critical attitude of community members towards environmental risks. The emphasis is placed on environmental protection and the prevention of health implications from pollution.

At this point, the instructions can be send to ChatGPT

Part 3 (analysis and description of knowledge flows)

Please note: It is helpful to provide ChatGPT with details where possible. So for instance when interested in knowledge flows in a particular range of articles that are part of a subchapter or specific section, it helps to mention this. So for example: "Please provide an analysis of knowledge flows for section 2, which covers articles 5 up to 12"

When working with larger sections (e.g. more than 10 articles), it can be helpful to cut the analysis in parts.

The instructions below can be provided to ChatGPT repeatedly (after a range or articles or section has been analyzed and a new section should be analyzed).

Note: In case further details or corrections are needed, instructions can be given to ChatGPT (see part 4)

In case of traditional policies (using articles)

For the document (DOCUMENT NAME), please provide an analysis of knowledge flows for articles ... up to … Use the format as specified before. Remember to avoid inferring or deducing the roles of any entities not directly cited in the text.

OR

For the document (DOCUMENT NAME), please provide an analysis of knowledge flows for article … Use the format as specified before. Remember to avoid inferring or deducing the roles of any entities not directly cited in the text.

For other documents (an example is provided, adapt according to structure)

For the document (DOCUMENT NAME), please provide an analysis of knowledge flows for sub-chapter ... Use the format as specified before. Remember to avoid inferring or deducing the roles of any entities not directly cited in the text.

Part 4 – asking further information, making corrections, providing guidance and particular tasks

Asking for further information / retrieving missed knowledge flows

- Could you provide more specific details on the different knowledge flows in article …?

- For the requirements / functions as specified in article … could you provide a summary?

Particular task: identify legal roles and responsibilities (of authorities)

For article … / section …, could you describe the different functions and responsibilities in one concise paragraph? If any, include functions or responsibilities in relation to water or wastewater.

- Optional (after getting output): Please provide an analysis of knowledge flows. Use the format as specified before. Remember to avoid inferring or deducing the roles of any entities not directly cited in the text.

OR

In the attached document (NAME DOCUMENT) different entities are discussed. I would like to have short descriptions of their role and/or responsibilities in environmental management. It concerns the following entities: …

- Optional (after getting output): Please provide an analysis of knowledge flows for these entities. Use the format as specified before. Remember to avoid inferring or deducing the roles of any entities not directly cited in the text.

Making corrections

When ChatGPT identifies senders or receivers that are not included in the text:

Please remember to indicate "not specified" for senders or receivers when these are not explicitly mentioned or referred to in the text. For example, … (give an example of a mistake).

Providing guidance

Helpful but no guarantee (with the state of progress in summer 2024). The instructions below can help in case you notice ChatGPT incorrectly analyses articles (confusing them or hallucinating). But issues may persist. Manual verification remains a necessity.

When ChatGPT has difficulties locating a section, article, or range of articles within a document (for example due to an unclear lay-out), provide it with the first and last phrase:

The section/range of articles starts with "…" and ends with "…"

OR

Please conduct an analysis of knowledge flows for article … The article starts with "…" and ends with "…"

OR

Please conduct an analysis of knowledge flows for article ... up to … The range starts with "…". It ends with "…".

Note: when issues persist, it can help to copy and paste the text directly into the chat. In that case, text parts should not be too long (e.g. specific articles or 2 to 3 articles at a time)

Supporting document: signal words for knowledge flows

1. Knowledge flows around documentation

1.1 Senders of knowledge

- A
  - apply to
- D
  - deliver to
- F
  - file before
- P
  - prepare for
  - present before/for
  - provide to
  - provided by
- R
  - report to
- S
  - submit to

1.2 Receivers of knowledge

- I
  - issued for
- O
  - obtain from
- R
  - request before
  - request from
  - required by
  - require from

2. Stakeholder interactions for decision-making

2.1 Senders of knowledge

- A
    - advocate for / advocacy
    - assist
- C
    - complain to
    - communicate with / communicate to
    - contribute to
- E
    - empower
- O
    - (provide) opinion (to)
- P
    - participate in / participation
    - provide (assistance, advice, input, opinions, …)
- S
    - seek from
    - suggest

2.2 Receivers of knowledge

- C
    - collaborate with
    - (in) consultation (with)
    - (in) cooperation with
    - coordinate with / (in) coordination (with)
- E
    - engage
- I
    - invite
    - involve
- p
    - (in) partnership with

3 Obtaining knowledge for making decisions

3.1 Receivers of knowledge

- D
  - demand from
- F
  - from primary information
  - from primary sources
- R
  - request before
  - request from
- S
  - seek (information, advice, suggestions, input, …) from

4. Expert assessment / official verification

4.1 Receivers of knowledge

- A
  - approve
  - assessed by
- C
  - certify
- E
  - endorse
- S
  - signed by
- V
  - verify before

5. Informing

5.1 Senders of knowledge

- A
  - (to raise) awareness
- D
  - disseminate
- E
  - encourage
  - explain
- O
  - outreach

6. Signal words for authorities issuing policies

6.1 Senders of knowledge

- D
  - delegate
  - determined by
- I
  - implement
  - issued by
- M
  - make regulations
- P
  - prepared by
  - provide a reference
- R
  - regulated by
  - regulated in

Supporting document: examples of knowledge flows

Examples of complete knowledge flows

Sender: communities

Receiver: person in charge of the business and/or activity

1. Communities directly affected as intended in article 29 have the right to submit suggestions, opinions and responses to business and/or activity plans with a period of 10 (ten) working days from the announcement as intended in article 28 paragraph (2) letter a.
2. Suggestions, opinions and responses as intended in paragraph (1) are submitted in writing to the person in charge of the business and/or activity.
3. In conveying suggestions, opinions and responses regarding the announcement of business plans and/or for activities as intended in paragraph (2), the public is required to include clear personal identity in accordance with population documents
4. Suggestions, opinions and responses from the public as intended in paragraph (3) can be in the form of:
    a. Descriptive information about environmental conditions in and around the location/site of the planned business and/or activity
    b. Local values that have the potential to be impacted by the planned business and/or activities to be carried out; and/or
    c. Community aspirations, desires and hopes related to business plans and/or activities

---

Sender: the public

Receiver: AMDAL document compiler

In this section, the AMDAL document compiler describes the information on the results of the community involvement process that is needed in the scoping process. It is important to remember that suggestions, opinions and responses received from the public must be processed before being used as input to the scoping process. This is because the suggestions, opinions and responses may be numerous and of various types and are not necessarily relevant to be studied in Amdal. Proof of the announcement and results of the public consultation can be attached. In detail, the information that must be explained includes key points that must be of concern to decision makers, namely what information is needed by decision makers regarding the results of community involvement, for example:

1) Descriptive information about the state of the environment around (“there are mangrove forests” or “many factories dump waste into river X”).

2) local values related to the proposed business plans and/or activities

3) local customs related to business plans and/or proposed activities

4) community aspirations related to proposed business plans and/or activities, including concerns about environmental changes that may occur ("don't let us run out of water" or "we don't like having outside

workers"); and expectations regarding improvements in the environment or welfare as a result of planned activities ("ask for clean water to be provided" or "ask local youth to be employed").

Sender: health workers, cadres, volunteers and/or the community

Receiver: the community

1) To achieve total sanitation conditions which include the 5 (five) STBM pillars as intended in Article 3 paragraph (2), after triggering, assistance is provided to the community.

2) Assistance as intended in paragraph (1) is carried out by health workers, cadres, volunteers and/or the community in implementing community work plans as intended in article 6.

Sender: experts, educational institutions, donor agencies, private sector and other relevant parties

Receivers: Government, provincial regional governments, and district/city regional governments

In supporting the implementation of STBM as intended in articles 9 to 12, the Government, provincial regional governments, and district/city regional governments can involve experts, educational institutions, donor agencies, the private sector and other relevant parties.

Sender: other people from inside or outside the village

Receiver: Village/district STBM Facilitator Team

1. Village/district STBM Facilitator Team consisting of at least volunteers, community leaders,

religious leaders, with the support of the village head, can be assisted by other people from

inside or outside the Village

Sender: public

Receiver: village midwives

2. Village midwives are expected to play a role as companions,

especially when there are questions from the public regarding medical

matters, and follow-up assistance as well as monitoring and evaluation

---

Sender: Environmental Agency

Receiver: person in charge of the Business and/or Activity

(5) In the event that the Business and/or Activity plan meets the provisions as referred to in paragraph (4) letter b, the person in charge of the Business and/or Activity requests direction from the Environmental Agency in accordance with its authority by attaching a summary of scientific considerations.

Sender: Environmental Due Test Team

Receiver: person responsible for the business and/or activity

(6) Based on the summary of scientific considerations submitted as intended in paragraph (5), the Environmental Due Test Team carries out studies and provides direction to the person responsible for the business and/or activity in the form of:

a. business plans and/or activities affecting the function of protected areas; or

b. business and/or activity plans that do not affect the function of the protected area

---

Sender: person in charge of the business and/or activity

Receiver: regional apparatus organization in charge of the provincial environment, or regional apparatus organization in charge of the regency/city environment

(2) In the event that the person in charge of the business and/or activity cannot carry out the screening properly independently, the person in charge of the business and/or activity submits a screening determination from the Central Environmental Agency, to the regional apparatus organization in charge of the provincial environment, or regional apparatus organization in charge of the regency/city environment in accordance with its authority.

Sender: communities

Receiver: person in charge of Business and/or Activities

(1) The Person in Charge of Business and/or Activities in preparing Amdal as intended in Article 21 paragraph (1) involves the community directly affected.

(2) involvement of communities directly affected as intended in paragraph (1) is carried out through:

a. announcement of business plans and/or activities; and

b. public consultation

(3) communities directly affected as intended in paragraph (1) have the right to submit suggestions, opinions and responses to business and/or activity plans within 10 (ten) working days from the announcement as intended in paragraph (2) letter a.

(4) Suggestions, opinions and responses intended in paragraph (3) are submitted in writing to the person in charge of the business and/or activity.

(5) Communities directly affected provide suggestions, opinions and responses to business and/or activity plans in public consultation as intended in paragraph (2) letter b.

(6) suggestions, opinions and responses to business and/or activity plans in public consultations as intended in paragraph (5) are recorded in the public consultation minutes.

(7) Involvement of communities directly affected as intended in paragraph (2) is carried out before the preparation of the Terms of Reference Form.

Sender: person in charge of the business and/or activity

Receivers: related agencies, community leaders, the public

(1) Before carrying out public consultation as intended in article 28 paragraph (2) letter b, the person in charge of the business and/or activity:

a. coordinates with related agencies and community leaders who will be involved in the public consultation process; and

b. inviting the public to be involved in public consultation

Sender 1: regency/city regional government

Receiver 1: provincial regional government

Sender 2: regency/city regional government

Receiver 2: the minister

(1) Water pollution load capacity and allocation of water pollution load as intended in article 9 paragraph (2) letter c is calculated by taking into account the report from the regency/city regional government consisting of:

a) Inventory of types and amounts of domestic wastewater in its administrative area;
b) Inventory of types and amounts of domestic wastewater processed in domestic wastewater treatment;
c) Inventory of domestic wastewater treatment technologies; and
d) Supervision of domestic wastewater processing, domestic wastewater treatment and fulfilment of domestic wastewater quality standards.

(2) Report as intended in paragraph (1) is submitted to the provincial regional government with a copy to the minister at least 1 (one) tome in 1 (one) year.

Sender: facilitator

Receiver: community

2. Break the ice

- Breaking the ice is done to create a friendly atmosphere between the facilitator and the community so that the community will be open to telling what is happening in the village.
- Breaking the ice can be done with entertaining games, easy for the community to play, involving many people.

Sender 1: person in charge of business and/or activities

Receiver 1: Minister, Governor, or Regent/Mayor

Sender 2: Minister, governor, or regent/mayor

Receiver 2: public

Sender 3: person in charge of the Business and/or Activity

Receiver 3: public

Sender 4: public

Receiver 4: not specified

1) The Person in charge of Business and/or Activities as intended in Article 86 submits a DELH or DPLH, which has been prepared through the Environmental Document Information System to the Minister, Governor, or Regent/Mayor in accordance with their authority.
2) The DELH or DPLH that has been prepared as intended in paragraph (1) is announced to the public through:
    a. Environmental document information system by the Minister, governor or regent/mayor in accordance with their authority; And
    b. Announcement at the Business and/or Activity location by the person in charge of the Business and/or Activity.
3) The announcement as intended in paragraph (2) contains information:
    a. Business and/or Activities along with evaluation of their Environmental Impact; And
    b. Environmental management and monitoring plan.
4) Based on the announcement as intended in paragraph (2) and paragraph (3), the public can provide suggestions, opinions and responses within a period of no later than 5 (five) working days from the announcement.

Sender: ministry

Receiver: Central government, provincial regional government, and regency/city regional government, person in charge of business and/or processing activities of domestic wastewater

This ministerial regulation aims to provide a reference regarding domestic wastewater quality standards to:

a) Provincial regional government in setting standards for stricter domestic wastewater quality
b) Central government, provincial regional government, and regency/city regional government, in issuing environmental permit, SPPL and/or wastewater discharge permit; and
c) Person in charge of business and/or processing activities of domestic wastewater in preparing domestic wastewater treatment plans, and preparing environmental documents.

Sender: provincial regional government

Receiver: provincial regional governments, district/city regional governments

Domestic wastewater quality standards set by provincial regional government as referred to in article 9, must be used by provincial regional governments and district/city regional governments in issuing environmental permit and/or wastewater disposal permit, unless other more stringent domestic wastewater quality standards are obtained through the results of environmental document reviews.

Sender 1: Minister

Sender 2: governor

Receiver: regent/mayor

1) Regency/city Water Quality Protection and Management Plan as intended in Article 117 letter c is prepared and determined by the regent/mayor.
2) The district/city Water Quality Protection and Management Plan as intended in paragraph (1) is applied to DAS within the district/city.
3) Determination of the district/city Water Quality Protection and Management Plan as intended in paragraph (2) is carried out after:
    a. Obtain technical considerations from the Minister; And
    b. Coordinate with the governor in the region.

Sender 1: other ministers
Receiver 1: Minister
Sender 2: Minister, regent/mayor
Receiver 2: governor
Sender 3: Minister, governor
Receiver 3: regent/mayor

(1) Water Quality Standards as intended in Article 113 paragraph (1) are prepared and determined by the Minister after coordinating with:

a. the minister who handles government affairs in the field of water resources;

b. the minister who handles government affairs in the field of energy and

mineral resources;

c. the minister who handles government affairs in the field of spatial planning; and/or

d. minister who handles government affairs in the forestry sector.

(2) Water Quality Standards as intended in Article 113 paragraph (1) are prepared and determined by the governor after:

a. obtain technical considerations from the Minister; And

b. coordinate with the regent/mayor.

(3) Water Quality Standards as intended in Article 113 paragraph (1) are prepared and determined by the regent/mayor after:

a. obtain technical considerations from the Minister; And

b. coordinate with the governor in the region.

Sender: the builder

Receiver: the community, community representatives

Step 3. Measures for social management, safety and health at work. During the construction works of the projects, the safety of the population must be guaranteed within the area of influence of the projects, including the surrounding populations, workers and end users, for which the following requirements must be met at least:

1. During the construction stage of the projects, the interaction with the community must be proactive and preventive.

2. Communication must be effective and contemplate a permanent and timely information process that allows generating changes in attitudes and ideologies in favor of the common good and strengthening conciliation mechanisms.

3. Communication channels must be appropriate between community representatives and the builder.

4. The meetings with the communities must have a structured program of topics.

5. Current regulations regarding the Occupational Health and Safety Management System (SG-SST) must be taken into account.

Sender: operators

Receiver: users (of the system)

The operators must carry out and document the inspections provided for in the operation and routine maintenance manuals and take the necessary actions for the optimal functioning of the systems. Likewise, you must make the updates that you consider pertinent in the manual, as long as they are

aimed at optimizing the operation of the systems. The record of all routine and preventive maintenance activities shall be documented.

In order to guarantee that the projects of the sector are effectively used in the populations and ensure the expected benefits, dissemination campaigns must be carried out for education, awareness and training for users, which must include at least topics related to water quality, responsibilities of users for the proper functioning of the systems, efficient use and saving of water, hygiene practices, climate change, proper management of liquid and solid waste.

Sender: person responsible for the activity

Receiver: Competent Environmental Authority

The person responsible for the activity may request before the competent Environmental Authority the exclusion of any parameter(s), as long as through material or mass balances and with the performance of the respective characterization it demonstrates that these are not present in the sewage. For this, the statistical analysis of the results of the characterizations and the information of the technical sheets of the raw materials and inputs used in the process must be carried out.

Sender: Attorney General of the Nation; Delegate for Environmental and Agrarian Affairs; the Ombudsman; the Minister of Environment and Sustainable Development; the General Directors of the other environmental authorities; the governors; the mayors; at least one hundred (100) people; three (3) non-profit entities

Receiver: environmental authority

Application. The holding of an environmental public hearing may be requested by the Attorney General of the Nation or the Delegate for Environmental and Agrarian Affairs, the Ombudsman, the Minister of Environment and Sustainable Development, the General Directors of the other environmental authorities, the governors, the mayors or at least one hundred (100) people or three (3) non-profit entities.

The request must be made to the environmental authority and contain the name and identification of the applicants, the address, the identification of the project, work or activity in respect of which the holding of the environmental public hearing is requested and the motivation thereof.

Sender: applicant/user

Receiver: environmental authority

Compliance Plan Requirement. If from the evaluation of the information coming from the characterization of the discharge, as well as from the documentation provided by the applicant, from the facts and circumstances deduced from the technical visits carried out by the competent environmental authority and from the technical report, it is concluded that it is not If it is feasible to grant the discharge permit, the competent environmental authority will require the user to submit a Compliance Plan, as

long as the discharge is not carried out in Class I water bodies referred to in article 2.2. 3.2.20.1 of this Decree.

The Compliance Plan must include the projects, works, activities and good practices that guarantee compliance with the discharge standard. Likewise, it must include its goals, its evaluation periods and its monitoring, management and results indicators with which the corresponding progress will be determined.

Sender: competent environmental authority

Receiver: interested parties

Publicity of the act ordered by the regulation. In order to notify the interested parties of the resolution by which the regulation of discharges is ordered, the competent environmental authority, within five (5) business days following the publication of the resolution, will proceed to:

1. Post for a term of ten (10) business days, in a public place at the headquarters of the competent environmental authority and on its website and in the corresponding City Hall or Police Inspectorate, a copy of the resolution.

2. Publish one (1) notice in one (1) newspaper with wide circulation in the region indicating the date, place and time of the technical visits. If there are facilities in the area, additionally this notice will be issued through the local radio station.

Sender: IDEAM (Institute of Hydrology, Meteorology and Environmental Studies)

Receiver: ethnic groups

Promotion and dissemination of the environmental experience of traditional cultures. The Institute of Hydrology, Meteorology and Environmental Studies -IDEAM-, will promote the development and dissemination of knowledge, values and technologies on environmental and natural resource management, of indigenous cultures and other ethnic groups, for which it will promote with the support of the Corporations and Institutes linked to the Ministries:

a) Programs, studies and research with the participation of ethnic groups.

b) Programs for the collection and rescue of ancestral experience and knowledge on the management of nature and its resources.

c) Diffusion and environmental education programs in support of various cultural groups in collaboration with ethnic education programs.

Sender: IDEAM

Receiver 1: the community

Receiver 2: environmental authorities

IDEAM must obtain, analyze, study, process and disseminate basic information on hydrology, hydrogeology, meteorology, basic geography on biophysical aspects, geomorphology. soils and vegetation cover for the management and use of the biophysical resources of the Nation and will be in charge of the establishment and operation of national meteorological and hydrological infrastructures to provide information, predictions, warnings and advisory services to the community.

This institute is responsible for monitoring the nation's biophysical resources, especially with regard to their contamination and degradation, necessary for decision-making by environmental authorities.

Sender 1: Ministry of Environment

Receiver 1: Regional Autonomous Corporation

Sender 2: Local community

Receiver 2: Regional Autonomous Corporation

Sender 3: Regional Autonomous Corporation

Receiver 3: Departments, Districts and Municipalities, territorial entities

Sender 4: authorities of indigenous communities, authorities of lands traditionally inhabited by black communities

Receiver 4: Regional Autonomous Corporation

Functions. The Regional Autonomous Corporations shall exercise the following functions:

1) Execute the national environmental policies, plans and programs defined by the law approving the National Development Plan and the National Investment Plan or by the Ministry of Environment, as well as those of a regional order that have been entrusted to it in accordance with the law, within the scope of its jurisdiction;

2) Exercise the function of highest environmental authority in the area of its jurisdiction, in accordance with the norms of a superior nature and in accordance with the criteria and guidelines drawn up by the Ministry of Environment;

3) promote and develop community participation in activities and programs for environmental protection, sustainable development and adequate management of renewable natural resources;

4) coordinate the process of preparing the environmental development plans, programs and projects that must be formulated by the different organizations and entities that are members of the National Environmental Management System (SINA) in the area of its jurisdiction and, in particular, advise the Department, Districts and Municipalities of their territory in the definition of environmental development plans and in their programs and projects regarding the protection of the environment and renewable natural resources, in order to ensure the harmony and coherence of the policies and actions adopted by the different territorial entities;

5) Advise territorial entities in the formulation of formal environmental education plans and execute informal environmental education programs, in accordance with the guidelines of the national policy

6) Advance, in coordination with the authorities of the indigenous communities and with the authorities of the lands traditionally inhabited by black communities, referred to in Law 70 of 1993, programs and projects for sustainable development and management, exploitation, use and conservation of renewable natural resources and the environment;

Sender 1: Ministry of Foreign Relations

Receiver 1: Ministry of Environment

The Ministry of Environment participates with the Ministry of Foreign Relations in the formulation of international policy on environmental matters and defines with it the instruments and procedures for cooperation in the protection of ecosystems in border areas; promotes relations with other countries in environmental matters and multilateral cooperation for the protection of natural resources and represents the National Government in the execution of International Treaties and Agreements on the environment and renewable natural resources;

Sender: MAVDT

Receiver 1: AARs

Receiver 2: national entities, AARs, territorial entities

In order to give continuity to the actions proposed by the PMAR, it is necessary to establish a follow-up program in charge of the DAPSBA of the MAVDT. This program must establish the mechanisms and periodicity for the collection of information and its systematization, at the national and regional levels. In turn, the information will feed the monitoring and evaluation indicators defined in the program.

The follow-up must guarantee permanent communication with contact points of the AARs, in order to have updated information on the actions that are carried out within the framework of the PMAR. The MAVDT will define the guidelines for the AARs to implement the regional management follow-up program.

Based on the information provided by the AARs, the MAVDT will present and disseminate with the national entities, AARs and territorial entities an annual progress report on the PMAR. This report will also contain recommendations to make adjustments in the execution of the PMAR.

Sender: Shehia Consultative Committee

Receiver 1: entrepreneurs, youth, elders, women and disabled persons

Receiver 2: community

Shehia Consultative Committee shall perform the following functions and powers:

(a) supervise and maintain the cooperative society within shehia;

(b) supervise the implementation of the employment plan within shehia;

(c) facilitate entrepreneurs, youth, elders, women and disabled persons in securing loans, credits and provisions of education on availability of reliable markets;

(d) educate the community about laws and regulations of the Local Government;

(e) sensitize the community on the importance of paying tax, fees and other charges for the shehia development

---

Sender: Local Government Development Committee

Receiver 1: District administration

Receiver 2: people in their area

Receiver 3: government agencies, Local Government Authorities, non-government organizations

The Local Government Development Committee shall hold meetings twice a year and may convene a special meeting when it deems necessary. The functions of Local Government Development Committee shall be:

(a) To implement local authority policies and to identify the challenges and advise the District administration on the best way to solve those problems and promote development of their respective areas;

(b) to monitor and assist the formulation of policies for Local Government Authorities in their areas for sustainable development;

(c) to mobilize people to participate, contribute and assist in the uses of resources and protection of the environment for sustainable development;

(d) to ensure that implementation strategies correspond to relevant policies and create awareness among the people in their areas; and

(e) to ensure and establish cooperation and coordination among government agencies, Local Government Authorities, and non-government organizations for creating an enabling environment and sustainable development

---

Sender 1: Relevant stakeholders

Receiver 1: Local Government Authority

Sender 2: Local Government Authority

Receiver 2: Authority

(1) Each Local Government Authority or any designated institution for waste shall manage both solid waste and waste water generated within their respective area of jurisdiction.

(2) Each Local Government Authority or designated institution for solid waste and waste water management, in consultation with relevant stakeholders, shall designate area for solid waste and waste water handling and disposal which shall be approved by the Authority.

(3) A person shall not handle or dispose any type of solid waste or waste water outside the designated area under this Act.

(4) A person who contravenes the provisions of this section commits an offence and upon conviction shall be liable to a fine of not less than one hundred thousand shillings and not exceeding five million shillings or imprisonment for a term of not less than three months and not exceeding two years or both fine and imprisonment.

(5) in addition to penalty imposed under this section, the court shall order to pay recovery expenses and compensation to the third party.

Sender: political leaders and faith-based groups

Receiver: General public/population

Enhanced involvement of political leaders and faith-based groups in order to mobilize popular support for efficiency in natural resources management.

Sender: National and local governments

Receiver: private sector and civil society organizations

i. National and local governments would be encouraged to build a strategic partnership with private sector and civil society organizations for effective environmental management, enforcement, and compliance.
ii. Public-private partnership for expansion and improvement of environmental services such as potable water supply, sewage disposal, efficient transport and efficient energy production would be promoted.
iii. Sector-specific environmental advisory committees involving public, private and civil society organizations would be established.

Sender: Minister

Receiver: Ministry responsible for water

The minister shall provide professional advice to the Ministry responsible for water to take all lawful, necessary and reasonably practicable measures for:

(a) preventing any pollution dangerous to public and environmental health or any supply of water which the public has right to use;

(b) purifying any such supply which has become so polluted; and

(c) taking measures include, if necessary, legal proceedings against any person so polluting or contaminating water source

Examples of incomplete knowledge flows

Sender: not specified

Receiver: society and business/private world

Strategies for increasing the role of society and the business/private world include:

1. Change behavior and increase public understanding of the importance of residential wastewater management;

2. Encourage participation from the business/private sector in implementing development and residential wastewater management.

This strategy is implemented with the following action plan:

1. Carry out outreach and campaigns regarding the importance of residential wastewater management

2. Providing assistance and training to the community in providing residential wastewater infrastructure and facilities;

3. Organizing pilot activities for the development of wastewater management infrastructure and facilities

---

Sender: not specified

Receiver: community

2. Increased sanitation needs

Component: increasing sanitation needs is a systematic effort to achieve changes in hygienic and sanitary behavior, in the form of:

a. triggering behavior change;

b. promotion and campaign to change hygiene and sanitation behavior

c. conveying messages through mass media and other communication media;

d. developing community commitment to change behavior;

e. facilitate the formation of community work teams; and

f. develop community/institutional reward mechanisms

Sender: person in charge of the business and/or activity

Receiver: not specified

(1) In carrying out public consultation, the person in charge of the business and/or activity shall convey relevant information:

a. description of business plans and/or activities

b. potential impacts that will arise from the initial identification of the person responsible for the business and/or activities include descreasing surface water quality, decreasing ambient air quality, environmental damage, public unrest, traffic disturbances, health disturbances, employment opportunities and business opportunities; and

c. environmental components that will be impacted by the planned business and/or activities

---

Sender: not specified

Receiver: Government, Regional Government, and stakeholders

1. Creation of a conducive environment

This component includes advocacy to the Government, Regional Government and stakeholders in developing a joint commitment to institutionalize development programs in rural sanitation, which is expected to produce:

a. Regional government commitment to provide resources to implement the STBM program stated in the letter of interest.
b. Regional policies and regional regulations regarding sanitation programs such as Regent's Decrees, regional regulations, Regional Medium Term Development Plans (RP JMD), Strategic Plans (Renstra), and others.
c. The formation of a coordinating institution that mainstreams the sanitation sector, which results in an increase in regional sanitation budgets as well as coordination of government and non-government resources.
d. The presence of facilitators, STBM trainers, and programs capacity increase.
e. A system for monitoring program performance results and learning management processes.

Sender: not specified

Receiver: Community

2. Increased sanitation needs

Component: Increasing sanitation needs is a systematic effort to achieve changes in hygienic and sanitary behavior, in the form of:

a. Triggering behavior change;
b. Promotion and campaign to change hygiene and sanitation behavior;
c. Conveying messages through mass media and other communication media
d. Developing community commitment to change behavior;
e. Facilitate the formation of community work teams; and

f. Develop community/institutional reward mechanisms.

Sender: minister (or head of relevant non-ministerial government agency)

Receiver: not specified

(1) The construction and provision of sanitation infrastructure must meet technical standards.

(2) Apart from having to meet the technical standards as intended in paragraph (1), the quality of the processed product of sanitation infrastructure must meet environmental quality standards.

(3) Technical standards and environmental quality standards as intended in paragraph (1) and paragraph (2) are determined by the minister or head of the relevant non-ministerial government agency.

Sender: minister, governor or regent/mayor

Receiver: not specified

(1) The RPPLH as intended in article 9 is prepared by the minister, governor or regent/mayor in accordance with their authority.

Sender: ministry

Receiver: not specified

(2) Further provisions regarding the types of business and/or activities that must be completed with an Amdal as intended in paragraph (1) are regulated by Ministerial regulations.

Sender: regent/mayor

Receiver: not specified

1) Regency/city Water Quality Protection and Management Plan as intended in Article 117 letter c is prepared and determined by the regent/mayor.
2) The district/city Water Quality Protection and Management Plan as intended in paragraph (1) is applied to DAS within the district/city.

Sender: official entities

Receiver: not specified

Determine the population directly or indirectly affected, as well as the population targeted or benefited by the execution of the project, calculated within the design period of the project. To establish a

baseline, reliable information must be sought from official entities related to the subject. This result must be included in the document mentioned in article 18 of this resolution.

Sender: community

Receiver: not specified

Formulation of community participation schemes in the processes of formulation and selection of technological alternatives, in the construction processes, start-up, operation and maintenance of the infrastructure.

Sender: populations served

Receiver: not specified

Social sustainability criteria. The development of projects in the sector must have studies related to the acceptability of the project, including the analysis of the sociocultural patterns of the populations involved in the face of the proposed alternatives. For this, the populations served must be involved during the planning stage of the project, in order to obtain timely information that can influence project decision-making, as well as the selection of the most favorable alternative.

Sender: populations served

Receiver: not specified

Social sustainability criteria. The development of projects in the sector must have studies related to the acceptability of the project, including the analysis of the sociocultural patterns of the populations involved in the face of the proposed alternatives. For this, the populations served must be involved during the planning stage of the project, in order to obtain timely information that can influence project decision-making, as well as the selection of the most favorable alternative.

Sender 1: Ministry of the interior

Sender 2: Incoder

Sender 3: Colombian institute of Anthropology and History

Receiver: not specified

The requirement to obtain the certifications that promote the protection of special communities and historical and cultural heritage must be verified, in accordance with current regulations:

1. Certification on the existence of ethnic communities in the area of influence of the project issued by the Ministry of the Interior. In the event of having the presence of this type of communities, the prior consultation process must be carried out, in accordance with the terms provided by the law.

2. Certificate of the existence or not of territories legally titled to indigenous reservations or collective titles belonging to Afro-Colombian communities in the area of influence of the project, issued by the Incoder.

3. Approval of an archaeological management plan or special management and protection plan by the Colombian Institute of Anthropology and History.

Sender: competent environmental authority

Origin of the regulation of discharges. The competent environmental authority, in order to obtain better control of the quality of water bodies, may regulate, ex officio or at the request of a party, the discharges that are carried out in these, in accordance with the results obtained in the Management Plan. of the Water Resource.

The objective of this regulation is that all the discharges made into the body of water make it possible to guarantee the current and potential uses of the same and the fulfillment of the quality objectives.

If the result of the Water Resource Management Plan determines the convenience and need to advance the regulation, the competent environmental authority will order it by resolution.

Said resolution will specify the date, place and time of the technical visits corresponding to the discharge regulation process.

Sender: regional autonomous corporations

Receiver: not specified

The regional autonomous corporations shall:

18) Order and establish the rules and guidelines for the management of hydrographic basins located within the area of its jurisdiction, in accordance with the superior provisions and national policies

---

Sender: not specified

Receiver: the agency

As a part of the scoping process the agency shall:

(4) hold an early scoping meeting or meetings which may be integrated with any other early planning meeting the agency has. Such a scoping meeting will often be appropriate when the impacts of a particular action are confined to specific sites.

Sender: Director of Environment

Receiver: not specified

On matters pertaining to the management of the environment, the Director of Environment shall:

(a) develop National Strategies and Guidelines of management of the environment;

(b) coordinate the implementation of National strategies and Guidelines of management of the environment;

(c) prepare and manage implementation of strategic environmental assessment according to section 48 of this act;

(d) recommend environmental standards;

(e) coordinate the implementation of international environmental agreements

---

Sender: Director of Environment

Receiver: not specified

(1) The Director of Environment shall promote environmental research that may contribute to proper environmental conservation and management.

(2) The Director of Environment shall store and utilize environmental research findings for the purpose of the implementation of this act.

Sender: the Authority

(1) the Authority shall oversee the execution of appropriate liquid waste management and disposal methods as prescribed in section 9 of this section.

(2) In executing its duty under subsection (1) of this section, the Authority shall:

(a) ensuring that sewage from cesspool and sludge from septic disposal of tanks are collected and transported by specified vehicles for liquid waste disposal;

(b) designating and ensure compliance with designated disposal ponds, sewage treatment facilities and sewer points;

(c) ensuring that before sewage is appropriately treated and prior to its discharge into water bodies or open land, the sewage will not increase the risk of infections or ecological disturbance and environmental degradation;

(d) making by-laws prescribing the treatment of hazardous and non-hazardous liquid waste;

(e) prescribing guidelines on standard gradient for storm water drains in order to prevent water stagnation, allow period cleaning of storm water drains to remove deposits and allow the inspection and removal of deposits in covered storm water drains, covers and appropriate trap chambers; and/or

(f) any other duty as deemed fit by the Authority

(3) for purposes of making decisions on suitability of any specific method of collection and treatment of liquid waste, the Authority shall engage findings from studies, assessments and surveys.
